# Leprosy elimination phase in Alagoas, 2001-2022: an ecological study

**DOI:** 10.1590/S2237-96222024v34e20240255.en

**Published:** 2025-04-11

**Authors:** Lucas Vinícius de Lima, Gabriel Pavinati, Rayssa Gysele Teixeira da Silva, Itanielly Gomes Queiroz, Gustavo Laine Araújo de Oliveira, Sandra Maria Barbosa Durães, Miguel Angel Aragón López, Kleydson Bonfim Andrade Alves, Francisco Beraldi-Magalhães, Gabriela Tavares Magnabosco

**Affiliations:** 1 Universidade Estadual de Maringá, Programa de Pós-Graduação em Enfermagem, Maringá, PR, Brazil; 2 Secretaria de Estado da Saúde de Alagoas, Gerência de Vigilância e Controle de Doenças Transmissíveis, Maceió, AL, Brazil; 3 Ministério da Saúde, Coordenação-Geral de Vigilância das Doenças em Eliminação, Brasília, DF, Brazil; 4 Organização Pan-Americana da Saúde, Coordenação de Doenças Transmissíveis e Determinantes Ambientais da Saúde, Brasília, DF, Brazil; 5 Universidade do Estado do Amazonas, Fundação de Medicina Tropical Doutor Heitor Vieira Dourado, Manaus, AM, Brazil

**Keywords:** Leprosy, Spatial Analysis, Public Health Surveillance, Ecological Studies, Time Series Studies, Lepra, Análisis Espacial, Vigilancia en Salud Pública, Estudios Ecológicos, Estudios de Series Temporales.

## Abstract

**Objective:**

To analyze the epidemiological scenario and leprosy elimination phase in the municipalities of Alagoas state, Brazil, from 2001 to 2022.

**Methods:**

This is an ecological study using surveillance data from the Notifiable Health Conditions Information System; detection and prevalence rates of the disease, along with absolute and relative frequencies, were used; temporal trends were evaluated using joinpoint regression, spatial autocorrelation was assessed with Moran’s index, and municipalities were classified according to the Leprosy Elimination Monitoring Tool (LEMT) proposed by the World Health Organization.

**Results:**

Between 2010 and 2022, there was a significant annual decrease of -2.89% (95% confidence interval [95%CI] -5.65; -0.91) in the prevalence rate, -4.43% (95%CI -6.56; -2.20) in the detection rate in the general population and -6.03% (95%CI -10.00; -1.46) in the detection rate in the population under 15 years of age; significant high-high clusters (p-value <0.050) were observed in the municipalities of Jacaré dos Homens, Pão de Açúcar and Carneiros; according to the LEMT in 2022: 7 (6.8%) municipalities were in phase 1 - up to interruption of transmission, 41 (40.2%) in phase 2 - up to disease elimination, 27 (26.5%) in phase 3 - post-elimination surveillance and 27 (26.5%) in phase 4 - non-endemic status.

**Conclusion:**

The data pointed to trends of reduction in the leprosy burden in the state, although some municipalities continue to show high rates; the use of LEMT with secondary data should be approached cautiously, as the absence of reported cases does not imply disease elimination in scenarios of underreporting and underdiagnosis.

## Resumen

**Objetivo:**

Analizar el panorama epidemiológico y la etapa de eliminación de la lepra en los municipios de Alagoas, Brasil, durante el período 2001-2022.

**Métodos:**

Estudio ecológico basado en datos de vigilancia obtenidos del Sistema de Información de Agravios de Notificación. Se utilizaron tasas de detección y prevalencia de la enfermedad, así como frecuencias absolutas y relativas. Se realizó un análisis de tendencias mediante regresión joinpoint, se aplicaron los índices de autocorrelación espacial de Moran y se clasificaron los municipios utilizando la herramienta Leprosy Elimination Monitoring Tool (LEMT), propuesta por la Organización Mundial de la Salud.

**Resultados:**

Entre 2010 y 2022, se observó una disminución anual significativa del -2,89% (intervalo de confianza del 95% [IC95%] -5,65; -0,91) en la tasa de prevalencia, del -4,43% (IC95% -6,56; -2,20) en la tasa de detección en la población general y del -6,03% (IC95% -10,00; -1,46) en la tasa de detección en la población menor de 15 años. Se identificaron conglomerados significativos de tipo alto-alto (p-valor<0,050) en los municipios de Jacaré dos Homens, Pão de Açúcar y Carneiros. Según la clasificación de la LEMT en 2022, 7 municipios (6,8%) se encontraban en la fase 1 - hasta la interrupción de la transmisión, 41 (40,2%) en la fase 2 - hasta la eliminación de la enfermedad, 27 (26,5%) en la fase 3 - vigilancia post-eliminación, y 27 (26,5%) en la fase 4 - estado no endémico.

**Conclusión:**

Los datos indican tendencias de reducción de la carga de lepra en el estado, aunque algunos municipios aún presentan tasas elevadas. El uso de la LEMT con datos secundarios debe manejarse con cautela, ya que la ausencia de casos no implica necesariamente la eliminación de la enfermedad en contextos de subnotificación y subdiagnóstico.

**Ethical aspects**

This research respected ethical principles, having obtained the following approval data:

This research respected ethical principles established by the National Health Council, and was exempt from review by the Research Ethics Committee of the Universidade Estadual de Maringá, as it involves secondary, publicly available, and de-identified data, in accordance with Article 26, Items III and V, of Resolution No. 674, dated May 6, 2022.

## Introduction

Leprosy remains a challenge, especially for countries with lower socioeconomic status and limited resources, such as Brazil ^1^ ^,^ ^2^. Although progress has been observed in the leprosy eliminating process, particularly through the Global Leprosy Strategy (2016-2020 and 2021-2030), proposed by the World Health Organization (WHO) ^2^, Brazil ranks second globally in the number of new cases of the disease ^1^ ^,^ ^3^.

In Brazil, the Ministry of Health has implemented National Strategies for Combating Leprosy (2019-2022 and 2024-2030) ^1^ ^,^ ^4^. The most recent proposal, developed through a participatory approach, aims to interrupt transmission in 99% of municipalities and eliminate the disease in 75% of them by 2030 ^4^. To achieve these goals, the following pillars were established: improving the management of leprosy programs, promoting early diagnosis through active case finding, and comprehensive care for individuals affected by the disease ^4^.

Between 2011 and 2021, 309,638 new cases of leprosy were reported in the country, representing an annual average of 28,000 cases ^5^. It is worth highlighting that the occurrence of the disease varies according to social, environmental, economic and demographic factors ^1^ ^,^ ^4^. In Brazil, in 2022, the occurrence was particularly concentrated in the North, Northeast and Midwest regions, with the states of Mato Grosso and Maranhão reporting the highest numbers of new cases ^4^.

A study identified clusters of high detection rates in the states of Pará, Tocantins, Maranhão and Mato Grosso; on the other hand, the states in the South and Southeast regions accounted for the lowest number of cases ^5^. In the Northeast region, the state of Alagoas stands out, with approximately 250 new cases annually ^6^. Although it is not a state with a high number of cases, compared to others in the same region, it has the second lowest human development index among Brazilian states ^7^.

The state of Alagoas aims to eliminate leprosy by 2030, in accordance with national and international guidelines. However, challenges persist, such as: weakness in case reporting, low engagement of family health teams in diagnosis and treatment, lack of health education initiatives, shortage of services and trained health professionals for diagnosis, and continued discrimination and stigma against affected people ^8^.

In 2023, WHO introduced definitions, criteria and indicators in the Leprosy Elimination Monitoring Tool (LEMT), a framework for assessing the interruption of the transmission chain and the phase of leprosy elimination ^9^ ^).^ LEMT provides a standardized approach to monitoring progress towards interruption of transmission and disease elimination, emphasizing evidence collection based on the WHO’s established milestones ^9^.

Internationally, LEMT has been used to assess the phases of leprosy elimination in Thailand, as illustrated by the WHO ^9^. In a search conducted in PubMed Central on October 5, 2024, using the strategy Leprosy Elimination Monitoring Tool, no studies were found on the tool’s application. Nationally, no scientific articles were found in the Virtual Health Library within the scope of this research using the same strategy.

Thus, the first step toward achieving the elimination goals proposed nationally and internationally by 2030 is the identification of priority areas to evaluate and redirect interventions, focusing on optimizing health actions and responses ^9^. This study is justified by its aim to analyze the epidemiological scenario and the phase of leprosy elimination in municipalities of Alagoas state, Brazil, from 2001 to 2022.

## Methods

### Design and background

This is an ecological study employing temporal and spatial analysis using data from 102 municipalities and from the state of Alagoas, located in Northeastern Brazil. Alagoas covers an area of 27,830.661 km2 and has a population of 3,127,683 people; the human development index is estimated at 0.684 ^7^ ^,^ ^10^. Its 102 municipalities are divided into ten health regions, enabling the organization of leprosy diagnostic and follow-up activities in primary health care.

### Participants and variables

In the first stage of the study, based on the panel, the timeframe from 2010 to 2022 was selected according to the availability of data on the date of access, November 17, 2023. Three indicators calculated by the Ministry of Health were extracted, and data on the inhabitants of each municipality obtained from the Brazilian Institute of Geography and Statistics, were used as the denominator. The following indicators were analyzed annually for the state and municipalities ^3^:

prevalence rate of cases under treatment (per 10,000 inhabitants): the number of cases under treatment in a given location on December 31 of the year of assessment, divided by the total population residing in the same location and period, and the result multiplied by 10,000;

detection rate of new cases in the general population (per 100,000 inhabitants): the number of new cases in a given location and diagnosed during the year of assessment, divided by the total population residing in the same location and period, and the result multiplied by 100,000; and

detection rate of new cases in the population under 15 years of age (per 100,000 inhabitants): the number of new cases in individuals under 15 years of age in a given location and diagnosed in the year of assessment, divided by the population under 15 years of age residing in the same location and period, and the result multiplied by 100,000.

For the second stage of the study, cases of leprosy reported as “new cases” diagnosed between 2001 and 2022 and residing in municipalities in Alagoas were analyzed. Records classified as “diagnosis errors” were excluded. This organization of the database allowed the creation of two variables, by municipality and year: (i) number of new cases of leprosy in the population aged 15 years and older and (ii) number of new cases of leprosy in the population under 15 years of age.

### Data source and measurement

Data from the Panel with Indicators and Basic Data on Leprosy in Brazilian Municipalities, compiled and made publicly available by the Brazilian Ministry of Health, and from the Notifiable Health Conditions Information System (Sistema Nacional de Informação de Agravos de Notificação - SINAN) ^6^, provided by the State Health Department of Alagoas, were used. The panel was used for the first stage of the study (temporal and spatial analysis). In this stage, the following epidemiological indicators of the disease were consulted:

prevalence rate of cases under treatment: low (<1.00/10,000 inhabitants), medium (1.00 to 4.99/10,000 inhabitants), high (5.00 to 9.99/10,000 inhabitants), very high (10.00 to 19.99/10,000 inhabitants) and hyperendemic (≥20.00/10,000 inhabitants);

detection rate of new cases in the general population: low (<2.00/100,000 inhabitants), medium (2.00 to 9.99/100,000 inhabitants), high (10.00 to 19.99/100,000 inhabitants), very high (20.00 to 39.99/100,000 inhabitants) and hyperendemic (≥40.00/100,000 inhabitants); and

detection rate of new cases in the population under 15 years of age: low (<0.50/100,000 inhabitants), medium (0.50 to 2.49/100,000 inhabitants), high (2.50 to 4.99/100,000 inhabitants), very high (5.00 to 9.99/100,000 inhabitants) and hyperendemic (≥10.00/100,000 inhabitants).

The SINAN database was employed for the second stage (analysis of the elimination phase). It is worth noting that the data from SINAN are used to feed the indicator panel, in line with the bottom-up data submission process. However, the panel contains pre-calculated indicators provided by the Ministry of Health relevant to the first stage of the study. In the second stage of the study, the period available on the panel was not compatible with the application of the LEMT.

The LEMT is an instrument proposed by the WHO for standardized monitoring of the leprosy elimination phase, organized into four sequential phases. Implementation of the tool at the subnational level encourages a bottom-up process in which each administrative area individually strives to meet the established milestones. For the operationalization of the LEMT, the following phases of municipal assessment were considered ^9^:

phase 1 - up to transmission interruption: the municipality remains in this phase until there are no new autochthonous cases in the population under 15 years of age for five consecutive years;

phase 2 - up to disease elimination: the municipality remains in this phase until there are no new autochthonous cases in the population aged 15 years and older for three consecutive years;

phase 3 - post-elimination surveillance: the municipality remains in this phase until there are no new autochthonous cases for at least ten consecutive years; and

phase 4 - non-endemic status: the municipality remains in this phase after sustaining the absence of new autochthonous cases recommended in the previous phase.

According to the epidemiological concept of autochthonous cases, these are cases where the origin of transmission can be traced to the same location where they occurred-that is, individuals who acquired the disease in their place of residence. Due to the lack of precise information on secondary data from SINAN, this study considered autochthonous cases as those registered in the population residing in the municipality.

Sporadic cases during phases 2 to 4 would need to be individually investigated to establish whether transmission occurred locally, thus classifying them as autochthonous. In such cases, the municipality would return to the previous phase. Furthermore, from phase 2 onwards, a municipality could regress if three or more new cases occurred, on average, for three consecutive years ^9^. In this study, only the second criterion was considered to revert to the phase due to the nature of the secondary data.

### Statistical methods

The analysis began with a temporal trend analysis of leprosy detection and prevalence rates in the state from 2010 to 2022. Joinpoint Regression Program, version 5.0.2, was used, applying the inflection point regression model, with the logarithmic transformation of the rates as the dependent variable and the years as the independent variable. First-order autocorrelation adjustment was applied and, and homoscedasticity was assumed due to the log transformation ^11^.

Final models were selected based on the lowest value of the weighted Bayesian information criterion. Using the empirical quantile method, the annual percentage change (APC) - rate changes at joinpoints; the average annual percentage change (AAPC) - geometric means of the APC; and 95% confidence intervals (95%CI) were calculated. Positive and negative APC/AAPC, whose 95%CI did not include the null value, indicated an increasing and decreasing trend, respectively (11).

In the first phase, the annual prevalence and detection rates, between 2010 and 2022, for each municipality in Alagoas were grouped by arithmetic mean to minimize random fluctuations. A publicly available shapefile of municipal and regional boundaries was used for analysis in the QGIS software, version 3.34.0. Thus, choropleth maps depicting spatial distributions were created based on endemicity classifications ^3^.

The global Moran’s index (I) was applied to determine the relationship (inverse or direct) of the average rate with itself in neighboring regions: an index closer to 1.00 indicated stronger autocorrelation. The first-order queen contiguity was employed and the significance was assessed using a 999 permutation test (p-value <0.050) in GeoDa, version 1.22. Local Moran’s index (Ii) was then calculated to identify the municipal values, with clustering patterns presented as ^12^:

high-high: municipality with a high rate, surrounded by municipalities with high rates;

low-low: municipality with a low rate, surrounded by municipalities with low rates;

high-low: municipality with a high rate, surrounded by municipalities with low rates; and

low-high: municipality with a low rate, surrounded by municipalities with high rates.

In the second phase, using data from 2001 to 2022, the municipalities were categorized by LEMT ^9^. Results were presented through four thematic maps, considering intentionally similar time intervals, namely: 2006, 2011, 2016 and 2022. As the first milestone (phase 1) requires at least five years, the initial period for presenting the choropleth maps was 2006. Subsequent intervals were defined by the authors for presentation purposes.

## Results

From 2010 to 2022, there was a declining trend of -2.89% (95%CI -5.65; -0.91) per year in the prevalence of leprosy in Alagoas, with a more pronounced decrease between 2010 and 2015 (APC -12.86; 95%CI -25.33; -6.98). Decreasing trends of -4.43% (95%CI -6.56; -2.20) per year in the detection rate of new cases of leprosy in the general population, and -6.03% (95%CI -10.00; -1.46) per year in the detection rate in the population under 15 years of age, were also noted (Table 1).

**1:** Annual percent changes (APC), average annual percent change (AAPC), and 95% confidence intervals (95%CI) for prevalence rates in the general population, detection in the general population, and detection in the population under 15 years of age in Alagoas. 2010-2022 (n=4,527)

Indicator	Period	APC (95%CI)	Trend	AAPC (95%CI)	Trend
Prevalence	2010-2015	-12.86 (-25.33; -6.98)	Decreasing	-2.89 (-5.65; -0.91)	Decreasing
	2015-2018	14.90 (-3.65; 24.14)	Stationary		
	2018-2022	-1.99 (-20.39; -5.85)	Decreasing		
Detection	2010-2022	-4.43 (-6.56; -2.20)	Decreasing	-4.43 (-6.56; -2.20)	Decreasing
Detection (<15 years old)	2010-2018	-1.36 (-5.68; 25.24)	Stationary	-6.03 (-10.00; -1.46)	Decreasing
2018-2022	-14.71 (-38.25; -5.23)	Decreasing

**Figure 1:**
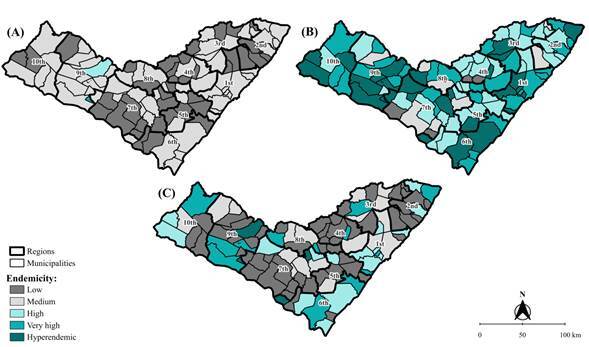
Endemic classification of average rates of prevalence (A), detection in the general population (B), and detection in the population under 15 years of age (C) for leprosy in Alagoas. 2010-2022 (n=4,527)

Higher average prevalence rates were observed in the municipalities of Palestina (6.06/10,000 inhabitants), Santana do Ipanema (2.95/10,000 inhabitants), and Delmiro Gouveia (2.43/10,000 inhabitants) (Figure 1A). The highest detection rates of new cases were also identified in the municipalities of Palestina (49.98/100,000 inhabitants and 16.59/100,000 inhabitants) and Santana do Ipanema (42.50/100,000 inhabitants and 14.98/100,000 inhabitants) (Figures 1B and 1C; Table 2).

**Table 2:** Municipal prevalence rates in the general population (per 10,000 inhabitants), detection rates in the general population (per 100,000 inhabitants), and detection rates in the population under 15 years of age (per 100,000 inhabitants) in Alagoas. 2010-2022 (n=4,527)

Municipality	Average rates		
	Prevalence	Detection	Detection (<15 years old)
Água Branca	0.46	4.56	2.30
Anadia	0.56	7.80	4.85
Arapiraca	1.18	13.21	1.89
Atalaia	0.50	7.33	2.11
Barra de Santo Antônio	0.84	7.45	3.45
Barra de São Miguel	2.17	31.27	6.67
Batalha	0.26	3.83	0.00
Belém	0.87	3.62	8.46
Belo Monte	0.00	0.00	0.00
Boca da Mata	0.24	3.43	0.00
Branquinha	1.01	13.08	2.19
Cacimbinhas	2.20	18.60	5.10
Cajueiro	0.47	14.79	7.73
Campestre	0.00	2.21	0.00
Campo Alegre	0.37	3.96	0.46
Campo Grande	0.26	1.75	0.00
Canapi	0.43	3.44	1.28
Capela	0.53	4.98	0.00
Carneiros	0.85	10.97	0.00
Chã Preta	0.42	4.15	0.00
Coité do Nóia	0.64	10.55	11.29
Colônia Leopoldina	0.29	5.21	1.05
Coqueiro Seco	1.20	16.07	4.68
Coruripe	1.16	15.09	3.35
Craíbas	0.87	9.40	0.99
Delmiro Gouveia	2.43	26.42	2.86
Dois Riachos	0.55	7.59	0.00
Estrela de Alagoas	0.81	8.97	1.53
Feira Grande	0.72	4.51	0.00
Feliz Deserto	0.67	5.07	0.00
Flexeiras	0.06	4.77	2.17
Girau do Ponciano	0.08	3.10	0.00
Ibateguara	0.41	2.52	0.00
Igaci	0.39	2.41	0.00
Igreja Nova	0.25	3.54	4.81
Inhapi	1.30	10.45	0.00
Jacaré dos Homens	1.02	10.10	0.00
Jacuípe	0.80	8.66	3.87
Japaratinga	1.18	6.35	0.00
Jaramataia	2.04	28.68	0.00
Jequiá da Praia	0.41	6.48	6.78
Joaquim Gomes	0.51	2.63	0.95
Jundiá	1.84	3.66	0.00
Junqueiro	0.22	3.79	0.00
Lagoa da Canoa	0.59	5.04	1.28
Limoeiro de Anadia	0.24	1.35	1.14
Maceió	0.78	9.73	2.10
Major Isidoro	1.50	13.61	2.71
Maragogi	1.11	15.11	5.46
Maravilha	0.58	2.46	0.00
Marechal Deodoro	0.74	8.13	3.31
Maribondo	0.97	9.06	0.00
Mar Vermelho	0.00	0.00	0.00
Mata Grande	0.89	7.08	6.29
Matriz de Camaragibe	0.22	3.75	0.00
Messias	0.24	3.68	2.96
Minador do Negrão	0.16	1.59	0.00
Monteirópolis	0.75	4.28	0.00
Murici	0.63	9.05	0.00
Novo Lino	0.61	3.06	0.00
Olho d'Água das Flores	1.80	16.96	6.29
Olho d'Água do Casado	0.60	6.83	0.00
Olho d'Água Grande	0.00	0.00	0.00
Olivença	0.54	6.13	0.00
Ouro Branco	0.68	7.48	2.21
Palestina	6.06	49.98	16.59
Palmeira dos Índios	0.67	7.79	1.25
Pão de Açúcar	1.75	25.94	9.57
Pariconha	0.29	12.43	2.62
Paripueira	0.89	7.21	0.00
Passo de Camaragibe	0.25	2.50	0.00
Paulo Jacinto	0.10	4.06	0.00
Penedo	1.22	17.86	5.12
Piaçabuçu	0.74	7.30	3.08
Pilar	1.83	22.45	3.39
Pindoba	0.00	0.00	0.00
Piranhas	0.66	7.16	0.00
Poço das Trincheiras	0.50	7.14	0.00
Porto Calvo	0.83	4.00	0.00
Porto de Pedras	0.80	6.40	2.67
Porto Real do Colégio	0.38	3.89	0.00
Quebrangulo	0.47	4.68	0.00
Rio Largo	1.22	12.17	1.11
Roteiro	0.92	3.47	0.00
Santa Luzia do Norte	0.53	8.54	7.88
Santana do Ipanema	2.95	42.50	14.98
Santana do Mundaú	0.49	5.59	0.00
São Brás	1.88	13.26	13.68
São José da Laje	0.89	4.59	2.25
São José da Tapera	0.72	13.45	6.27
São Luís do Quitunde	0.09	0.91	0.00
São Miguel dos Campos	0.27	3.27	0.49
São Miguel dos Milagres	0.48	3.80	0.00
São Sebastião	0.25	3.24	0.00
Satuba	0.96	11.17	4.33
Senador Rui Palmeira	0.97	7.92	0.00
Tanque d'Arca	0.25	3.71	0.00
Taquarana	0.47	4.26	3.03
Teotônio Vilela	0.97	13.43	1.71
Traipu	0.48	1.94	0.00
União dos Palmares	1.33	22.14	7.50
Viçosa	0.74	4.77	0.00

**Figure 2:**
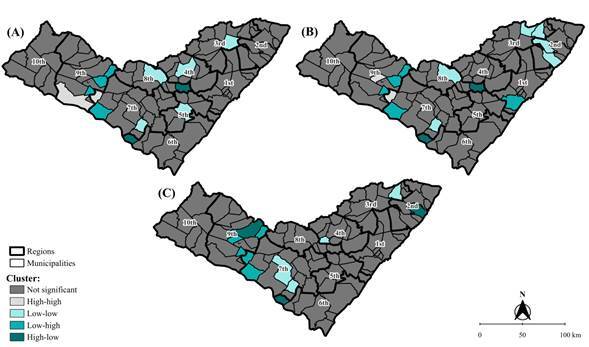
Spatial autocorrelation of average rates of prevalence (A), detection in the general population (B), and detection in the population under 15 years of age (C) for leprosy in Alagoas. 2010-2022 (n=4,527)

Based on the global Moran’s index, no autocorrelation was observed for prevalence rates (I: -0.01; p-value 0.483) and detection in the general population (I: 0.01; p-value 0.297) and among individuals under 15 years of age (I: -0.07; p-value 0.146). When applying the local indicator, high-high clusters were identified for the municipalities of Jacaré dos Homens and Pão de Açúcar for prevalence rates (Figure 2A); and in Carneiros and Jacaré dos Homens for detection rates in the general population (Figure 2B).

**Figure 3:**
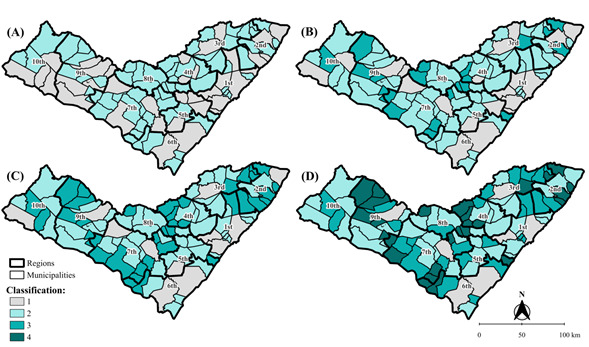
Classification by the Leprosy Elimination Monitoring Tool of leprosy elimination phase in Alagoas. 2006 (A), 2011 (B), 2016 (C), and 2022 (D)

The categorization of municipalities by elimination phases was presented in the maps for 2006 (Figure 3A), 2011 (Figure 3B), 2016 (Figure 3C) and 2022 (Figure 3D). By the end of the analyzed period, it was identified that: 7 (6.8%) municipalities were in phase 1; 41 (40.2%) were in phase 2;27 (26.5%) were in phase 3; and 27 (26.5%) were in phase 4. The data for each municipality can be individually visualized in the LEMT spreadsheet (Supplementary Table 1).

## Discussion

The state of Alagoas is committed to achieving the elimination of leprosy as a public health problem by 2030, showing a decreasing trend in prevalence rates, detection rates in the general population and detection rates in the population under 15 years of age from 2010 to 2022. Among the municipalities evaluated between 2001 and 2022, 28 stood out as being in the post-elimination phase, and 27 were classified as non-endemic.

The reduction in the magnitude and incidence of leprosy may reflect the strengthening of strategies, such as: increased early diagnosis through decentralization to primary care; promotion of preventive educational actions among the population; expansion of family health coverage to enable the early identification of at-risk individuals; and strengthening of active case finding, particularly among social contacts and family members of affected individuals ^13^ ^-^ ^18^.

Although the downward trend in leprosy indicators aligns with other national studies, the disease shows high detection rates in regions of poverty and social inequality ^13^ ^-^ ^15^ ^,^ ^18^ . Ecological studies have revealed that the population’s poor living conditions, such as low per capita household income, low education level and high number of residents per household, acted as barriers to access to diagnosis and increased the risk of leprosy ^19^ ^-^ ^21^.

In the state of Alagoas, despite the reduction in prevalence and detection indicators, some municipalities, including Palestina, Santana do Ipanema and Delmiro Gouveia, remained hyperendemic. In 2010, these municipalities had the following human development index: 0.558, 0.591 and 0.612 ^7^. Furthermore, the average Gini index for these municipalities was 0.573, exceeding the value for the state, which was 0.526, indicating greater income inequality ^7^.

In addition, the municipalities of Jacaré dos Homens, Pão de Açúcar and Carneiros showed clusters with higher leprosy burden, according to the autocorrelation assessment. These municipalities are located in the ninth health region, which suggests that this area concentrates most municipalities with high detection and prevalence rates. This indicates the need for targeted interventions in these territories, aiming to optimize the state’s response.

In 2015, projections indicated that elimination targets could be achieved by 2020 in Brazil, India and Indonesia. However, leprosy remained a problem in endemic regions of these countries ^22^, particularly due to resource prioritization challenges, leaving the most vulnerable populations at increased risk ^23^. In Brazil, a cohort of 23,899,942 individuals revealed that lower income and education levels were associated with an up to twofold increase in the incidence of the disease ^24^.

Conversely, areas with better access to health services may show higher detection rates for leprosy cases ^25^. Thus, taking into consideration socioeconomic and programmatic issues, especially through studies that allow the identification of local and regional specificities associated with the epidemiological context, can help identify priority areas for evaluating strategies and directing new interventions ^26^ ^,^ ^27^.

While low prevalence and detection rates may indicate progress in disease control in some regions, it is worth highlighting that improved access to health services remains a crucial factor associated with the increase in the leprosy detection rate ^25^. Thus, the absence of leprosy cases in municipalities undergoing elimination process must be cautiously interpreted, as it may result from weaknesses in the health system regarding case detection/notification.

Although LEMT allows for the occurrence of sporadic new cases in phases 3 and 4 ^9^, individual analyses are necessary for each situation, given that factors such as the grade of physical disability at diagnosis and the contact evaluation to identify epidemiological links should be considered as potential confounding factors in defining “sporadic”. Consequently, state-level coordination with municipalities is indispensable to ensure greater confidence in using the LEMT.

However, it is noteworthy that the assessment of the phases of leprosy elimination in this study included two distinct parameters ^9^. This suggests that municipalities in more advanced phases may indeed show effective progress in disease control. However, it is worth emphasizing the need to strengthen disease surveillance systems - especially the SINAN system -, which are crucial for monitoring trends and patterns in leprosy cases using secondary data ^28^.

Regarding leprosy elimination, in addition to reducing the number of cases, efforts to combat discrimination and prevent disabilities are emphasized ^29^. The physical, psychological and social domains of the people affected are among the most compromised aspects of their quality of life ^30^. Thus, despite the epidemiological issues highlighted in this study, it is essential to evaluate living conditions across different regions of the country to monitor the elimination response.

This study has limitations, including the use of secondary data, which is subject to underreporting; reliance of national datasets, as the indicator panel’s database may differ from state databases due to qualification processes; assessment during the COVID-19 pandemic, which may have impacted detection/notification quality between 2020 and 2022 ^5^; and inability of individual assessment of autochthonous/sporadic cases due to the exclusive use of secondary data.

Findings indicated a significant reduction in the prevalence and detection of leprosy in Alagoas, suggesting the strengthening of health strategies, such as early diagnosis and active case finding. However, the persistence of hyperendemic areas, especially in regions of social inequality, highlights the need for focused interventions. These actions should prioritize access to health services, surveillance and measures to combat discrimination, which are essential to achieve elimination.

Furthermore, isolated use of LEMT should be approached with caution, as the absence of cases does not imply elimination in the territories; in these situations, parameters such as underdiagnosis and underreporting, evidenced by high grade of physical disability at diagnosis, lack of active case finding, and weaknesses in testing and surveillance services, should be consider. In addition, autochthonous and sporadic cases require individual analysis to assess the epidemiological link and community transmission.

It is worth highlighting that the validation of methods and results with healthcare professionals and federal and state managers was crucial for understanding certain municipal dynamics that deviated from the expected pattern in the process of elimination. In this context, the use of LEMT should consider the path from micro level to macro level, as this coordination can enhance the use of the tool and improve the interpretation of results for each region.

## Data Availability

The databases used in the research are available at https://datasus.saude.gov.br/acesso-a-informacao/casos-de-hanseniase-desde-2001sinan/ and http://indicadoreshanseniase.aids.gov.br/.
