# Supplementary material for: Leprosy elimination phase in Alagoas, 2001-2022: an ecological study
Source: Epidemiol Serv Saude. 2025 Apr 11;34:e20240255. doi: 10.1590/S2237-96222024v34e20240255.en (PMC11998656; doi:10.1590/S2237-96222024v34e20240255.en)
Supplement: Supplementary file 1 [file 2237-9622-ress-34-e20240255-en-sup.pdf]

Supplementary Table 1. Classification by the Leprosy Elimination Monitoring Tool of the leprosy elimination phase in Alagoas. 2001-2022

Leprosy Elimination Monitoring Tool

- **Phase 1 – up to transmission interruption**
- **Phase 2 – up to disease elimination**
- **Phase 3 – post-elimination surveillance**
- **Phase 4 – non-endemic status**
- **Sporadic cases in adults**
- **Sporadic cases in children**
- **Average of three cases over three consecutive years**

Municipality	Cases	2001	2002	2003	2004	2005	2006	2007	2008	2009	2010	2011	2012	2013	2014	2015	2016	2017	2018	2019	2020	2021	2022
Água Branca	<15 years	0	0	0	0	0	0	0	0	0	0	0	0	0	1	1	0	0	0	0	0	0	0
	Adult	0	0	0	1	0	2	1	0	0	0	1	3	1	1	1	0	0	1	1	0	1	0
	Total	0	0	0	1	0	2	1	0	0	0	1	3	1	2	2	0	0	1	1	0	1	0
Anadia	<15 years	1	0	0	0	0	0	0	0	0	0	0	0	1	0	0	0	2	0	0	0	0	0
	Adult	1	1	1	3	3	2	1	1	1	4	1	3	1	4	0	0	0	1	1	1	0	0
	Total	2	1	1	3	3	2	1	1	1	4	1	3	2	4	0	0	2	1	1	1	0	0
Arapiraca	<15 years	3	5	4	1	5	4	2	2	2	2	2	1	0	1	0	0	1	1	2	0	2	1
	Adult	33	25	37	28	30	43	36	24	22	28	30	62	36	23	28	18	25	28	29	18	23	24
	Total	36	30	41	29	35	47	38	26	24	30	32	63	36	24	28	18	26	29	31	18	25	25
Atalaia	<15 years	0	0	0	0	0	1	0	0	0	0	2	0	1	0	1	0	0	1	0	0	0	0
	Adult	0	1	1	2	3	4	3	5	9	6	2	4	7	3	6	0	4	4	3	1	1	1
	Total	0	1	1	2	3	5	3	5	9	6	4	4	8	3	7	0	4	5	3	1	1	1
Barra de Santo Antônio	<15 years	0	1	0	0	0	0	0	1	0	0	0	1	0	0	0	0	0	0	0	0	0	1
	Adult	2	1	1	1	1	1	0	2	1	0	1	3	0	1	0	1	0	1	1	1	1	2
	Total	2	2	1	1	1	1	0	3	1	0	1	4	0	1	0	1	0	1	1	1	1	3
Barra de São Miguel	<15 years	0	0	0	1	1	0	0	2	0	0	0	0	0	0	1	1	0	0	0	0	0	0
	Adult	3	0	3	3	4	2	0	2	2	1	3	5	2	2	4	4	0	3	4	1	1	1
	Total	3	0	3	4	5	2	0	4	2	1	3	5	2	2	5	5	0	3	4	1	1	1

Continued

Supplementary Table 1. Classification by the Leprosy Elimination Monitoring Tool of the leprosy elimination phase in Alagoas. 2001-2022

Leprosy Elimination Monitoring Tool

- **Phase 1 – up to transmission interruption**
- **Phase 2 – up to disease elimination**
- **Phase 3 – post-elimination surveillance**
- **Phase 4 – non-endemic status**
- **Sporadic cases in adults**
- **Sporadic cases in children**
- **Average of three cases over three consecutive years**

Municipality	Cases	2001	2002	2003	2004	2005	2006	2007	2008	2009	2010	2011	2012	2013	2014	2015	2016	2017	2018	2019	2020	2021	2022
Batalha	<15 years	0	0	0	0	0	0	0	0	0	0	0	0	0	0	0	0	0	0	0	0	0	0
	Adult	1	0	1	1	0	1	2	1	1	2	1	0	1	1	0	0	0	1	1	1	1	0
	Total	1	0	1	1	0	1	2	1	1	2	1	0	1	1	0	0	0	1	1	1	1	0
Belém	<15 years	0	0	0	0	0	0	0	0	0	0	0	0	0	0	0	0	0	0	0	1	0	0
	Adult	0	0	0	0	0	0	0	0	0	0	0	0	0	0	0	0	0	0	0	0	1	0
	Total	0	0	0	0	0	0	0	0	0	0	0	0	0	0	0	0	0	0	0	1	1	0
Belo Monte	<15 years	0	0	0	0	0	0	0	0	0	0	0	0	0	0	0	0	0	0	0	0	0	0
	Adult	0	0	0	1	1	0	0	0	1	0	0	0	0	0	0	0	0	0	0	0	0	0
	Total	0	0	0	1	1	0	0	0	1	0	0	0	0	0	0	0	0	0	0	0	0	0
Boca da Mata	<15 years	1	0	0	0	0	0	0	0	0	0	0	0	0	0	0	0	0	0	0	0	0	0
	Adult	4	0	8	0	1	1	0	0	1	6	0	0	2	0	0	2	1	0	0	0	0	0
	Total	5	0	8	0	1	1	0	0	1	6	0	0	2	0	0	2	1	0	0	0	0	0
Branquinha	<15 years	0	0	0	0	0	0	0	0	1	0	0	0	0	1	0	0	0	0	0	0	0	0
	Adult	1	1	3	0	4	2	1	2	1	1	3	1	2	1	5	1	1	0	2	0	2	1
	Total	1	1	3	0	4	2	1	2	2	1	3	1	2	2	5	1	1	0	2	0	2	1
Cacimbinhas	<15 years	0	0	0	0	0	0	0	0	0	1	0	0	0	0	0	0	0	1	0	0	0	0
	Adult	0	0	1	0	1	0	0	0	0	0	0	1	2	2	2	2	1	6	4	2	0	3
	Total	0	0	1	0	1	0	0	0	0	1	0	1	2	2	2	2	1	7	4	2	0	3

Continued

Supplementary Table 1. Classification by the Leprosy Elimination Monitoring Tool of the leprosy elimination phase in Alagoas. 2001-2022

Leprosy Elimination Monitoring Tool

- **Phase 1 – up to transmission interruption**
- **Phase 2 – up to disease elimination**
- **Phase 3 – post-elimination surveillance**
- **Phase 4 – non-endemic status**
- **Sporadic cases in adults**
- **Sporadic cases in children**
- **Average of three cases over three consecutive years**

Municipality	Cases	2001	2002	2003	2004	2005	2006	2007	2008	2009	2010	2011	2012	2013	2014	2015	2016	2017	2018	2019	2020	2021	2022
Cajueiro	<15 years	0	1	0	0	1	0	0	0	0	0	0	0	0	0	0	2	3	0	0	0	0	0
	Adult	1	3	0	3	1	0	0	2	0	0	2	2	0	2	7	4	7	6	1	3	3	1
	Total	1	4	0	3	2	0	0	2	0	0	2	2	0	2	7	6	10	6	1	3	3	1
Campestre	<15 years	0	0	0	0	0	0	0	1	0	0	0	0	0	0	0	0	0	0	0	0	0	0
	Adult	0	0	0	1	0	0	1	0	1	0	0	0	0	1	0	0	0	1	0	0	0	0
	Total	0	0	0	1	0	0	1	1	1	0	0	0	0	1	0	0	0	1	0	0	0	0
Campo Alegre	<15 years	0	0	0	0	0	0	0	0	0	0	0	1	0	0	0	0	0	0	0	0	0	0
	Adult	2	0	1	2	2	4	4	1	2	6	0	2	0	3	2	1	3	3	1	4	0	2
	Total	2	0	1	2	2	4	4	1	2	6	0	3	0	3	2	1	3	3	1	4	0	2
Campo Grande	<15 years	0	0	0	0	0	0	0	0	0	0	0	0	0	0	0	0	0	0	0	0	0	0
	Adult	1	0	0	0	0	0	0	0	0	0	0	0	1	0	0	0	0	0	0	1	0	1
	Total	1	0	0	0	0	0	0	0	0	0	0	0	1	0	0	0	0	0	0	1	0	1
Canapi	<15 years	0	0	0	0	0	0	0	0	0	0	0	0	0	0	1	0	0	0	0	0	0	0
	Adult	0	0	0	1	1	0	0	0	0	0	0	0	0	2	1	0	1	0	2	0	1	0
	Total	0	0	0	1	1	0	0	0	0	0	0	0	0	2	2	0	1	0	2	0	1	0
Capela	<15 years	0	0	0	0	0	0	0	0	0	0	0	0	0	0	0	0	0	0	0	0	0	0
	Adult	0	1	0	1	1	0	1	1	0	1	2	1	0	0	2	0	0	2	0	0	2	1
	Total	0	1	0	1	1	0	1	1	0	1	2	1	0	0	2	0	0	2	0	0	2	1

Continued

Supplementary Table 1. Classification by the Leprosy Elimination Monitoring Tool of the leprosy elimination phase in Alagoas. 2001-2022

Leprosy Elimination Monitoring Tool

- **Phase 1 – up to transmission interruption**
- **Phase 2 – up to disease elimination**
- **Phase 3 – post-elimination surveillance**
- **Phase 4 – non-endemic status**
- **Sporadic cases in adults**
- **Sporadic cases in children**
- **Average of three cases over three consecutive years**

Municipality	Cases	2001	2002	2003	2004	2005	2006	2007	2008	2009	2010	2011	2012	2013	2014	2015	2016	2017	2018	2019	2020	2021	2022
Carneiros	<15 years	0	0	0	0	0	0	0	0	0	0	0	0	0	0	0	0	0	0	0	0	0	0
	Adult	1	0	0	0	1	0	0	0	0	0	0	0	1	0	2	3	1	0	1	0	2	0
	Total	1	0	0	0	1	0	0	0	0	0	0	0	1	0	2	3	1	0	1	0	2	0
Chã Preta	<15 years	0	0	0	0	1	0	0	0	0	0	0	0	0	0	0	0	0	0	0	0	0	0
	Adult	0	0	1	0	0	0	0	0	1	0	0	0	0	1	2	0	0	1	0	0	0	0
	Total	0	0	1	0	1	0	0	0	1	0	0	0	0	1	2	0	0	1	0	0	0	0
Coité do Nóia	<15 years	0	0	0	0	0	0	0	0	0	0	0	1	0	0	0	0	0	2	0	0	0	0
	Adult	0	1	1	0	1	0	0	0	1	1	0	1	0	0	2	0	0	4	2	0	1	0
	Total	0	1	1	0	1	0	0	0	1	1	0	2	0	0	2	0	0	6	2	0	1	0
Colônia Leopoldina	<15 years	0	0	0	0	0	0	0	1	0	1	0	0	0	0	0	0	0	0	0	0	0	0
	Adult	0	1	0	0	1	1	0	0	1	3	1	1	1	0	1	0	0	0	1	1	3	1
	Total	0	1	0	0	1	1	0	1	1	4	1	1	1	0	1	0	0	0	1	1	3	1
Coqueiro Seco	<15 years	0	0	0	0	0	0	1	0	0	0	0	0	0	0	1	0	0	0	0	0	0	0
	Adult	0	1	3	0	1	0	0	2	0	3	1	2	4	1	0	0	0	0	0	1	1	1
	Total	0	1	3	0	1	0	1	2	0	3	1	2	4	1	1	0	0	0	0	1	1	1
Coruripe	<15 years	1	0	0	0	3	1	0	1	1	1	1	0	0	2	0	0	0	1	1	1	0	0
	Adult	5	8	8	9	12	7	12	6	12	10	6	8	8	5	10	5	8	7	5	3	15	14
	Total	6	8	8	9	15	8	12	7	13	11	7	8	8	7	10	5	8	8	6	4	15	14

Continued

Supplementary Table 1. Classification by the Leprosy Elimination Monitoring Tool of the leprosy elimination phase in Alagoas. 2001-2022

Leprosy Elimination Monitoring Tool

- **Phase 1 – up to transmission interruption**
- **Phase 2 – up to disease elimination**
- **Phase 3 – post-elimination surveillance**
- **Phase 4 – non-endemic status**
- **Sporadic cases in adults**
- **Sporadic cases in children**
- **Average of three cases over three consecutive years**

Municipality	Cases	2001	2002	2003	2004	2005	2006	2007	2008	2009	2010	2011	2012	2013	2014	2015	2016	2017	2018	2019	2020	2021	2022
Craíbas	<15 years	0	0	0	0	0	0	0	0	0	1	0	0	0	0	0	0	0	0	0	0	0	0
	Adult	0	2	7	6	7	3	1	3	1	4	4	3	3	3	3	3	0	2	1	0	3	1
	Total	0	2	7	6	7	3	1	3	1	5	4	3	3	3	3	3	0	2	1	0	3	1
Delmiro Gouveia	<15 years	1	0	1	2	1	1	0	0	0	1	0	1	0	0	0	0	0	0	1	1	0	2
	Adult	8	8	32	17	22	20	15	14	15	14	19	20	12	13	17	16	7	14	7	9	13	12
	Total	9	8	33	19	23	21	15	14	15	15	19	21	12	13	17	16	7	14	8	10	13	14
Dois Riachos	<15 years	0	1	0	0	1	0	0	0	3	0	0	0	0	0	0	0	0	0	1	0	0	0
	Adult	0	0	1	0	1	1	2	1	10	1	1	2	2	0	0	1	2	1	1	0	0	0
	Total	0	1	1	0	2	1	2	1	13	1	1	2	2	0	0	1	2	1	2	0	0	0
Estrela de Alagoas	<15 years	0	0	0	0	0	0	0	0	0	0	0	0	0	1	0	0	0	0	0	0	0	0
	Adult	1	2	1	3	2	4	3	1	0	1	1	0	2	4	3	0	1	1	1	2	0	1
	Total	1	2	1	3	2	4	3	1	0	1	1	0	2	5	3	0	1	1	1	2	0	1
Feira Grande	<15 years	0	0	0	0	0	0	0	0	0	0	0	0	0	0	0	0	0	0	0	0	0	0
	Adult	1	0	0	0	3	0	0	1	0	0	1	1	0	0	0	1	0	1	1	4	2	2
	Total	1	0	0	0	3	0	0	1	0	0	1	1	0	0	0	1	0	1	1	4	2	2
Feliz Deserto	<15 years	0	0	0	0	0	0	0	1	0	0	0	0	0	0	0	0	0	0	0	0	0	0
	Adult	3	2	1	0	0	1	1	0	0	1	0	1	1	0	0	0	0	0	0	0	0	0
	Total	3	2	1	0	0	1	1	1	0	1	0	1	1	0	0	0	0	0	0	0	0	0

Continued

Supplementary Table 1. Classification by the Leprosy Elimination Monitoring Tool of the leprosy elimination phase in Alagoas. 2001-2022

Leprosy Elimination Monitoring Tool	
	Phase 1 – up to transmission interruption
	Phase 2 – up to disease elimination
	Phase 3 – post-elimination surveillance
	Phase 4 – non-endemic status
	Sporadic cases in adults
	Sporadic cases in children
	Average of three cases over three consecutive years

Municipality	Cases	2001	2002	2003	2004	2005	2006	2007	2008	2009	2010	2011	2012	2013	2014	2015	2016	2017	2018	2019	2020	2021	2022
Flexeiras	<15 years	0	0	0	1	0	0	0	0	0	0	0	0	0	0	0	0	1	0	0	0	0	0
	Adult	2	2	2	0	1	1	1	0	2	1	0	0	0	1	2	1	0	1	0	0	0	0
	Total	2	2	2	1	1	1	1	0	2	1	0	0	0	1	2	1	1	1	0	0	0	0
Girau do Ponciano	<15 years	0	0	0	0	0	0	0	0	0	0	0	0	0	0	0	0	0	0	0	0	0	0
	Adult	0	0	1	2	2	1	0	1	1	0	1	4	0	2	4	2	0	1	1	0	1	0
	Total	0	0	1	2	2	1	0	1	1	0	1	4	0	2	4	2	0	1	1	0	1	0
Ibateguara	<15 years	0	0	1	0	0	1	0	1	0	0	0	0	0	0	0	0	0	0	0	0	0	0
	Adult	5	2	5	2	1	1	0	1	0	1	0	0	2	0	0	0	0	0	0	0	1	2
	Total	5	2	6	2	1	2	0	2	0	1	0	0	2	0	0	0	0	0	0	0	1	2
Igaci	<15 years	0	0	0	0	0	0	0	0	0	0	0	0	0	0	0	0	0	0	0	0	0	0
	Adult	1	1	0	0	1	1	0	2	1	1	2	3	0	1	1	0	0	0	0	0	0	0
	Total	1	1	0	0	1	1	0	2	1	1	2	3	0	1	1	0	0	0	0	0	0	0
Igreja Nova	<15 years	0	0	0	0	0	0	0	0	0	1	1	0	0	0	0	0	0	0	0	0	1	0
	Adult	0	0	5	1	0	4	2	1	2	1	1	0	2	1	0	0	0	0	0	0	1	1
	Total	0	0	5	1	0	4	2	1	2	2	2	0	2	1	0	0	0	0	0	0	2	1
Inhapi	<15 years	0	0	1	0	0	0	1	0	0	0	0	0	0	0	0	0	0	0	0	0	0	0
	Adult	2	0	0	1	0	0	1	3	0	5	1	7	1	2	0	0	2	1	1	3	2	4
	Total	2	0	1	1	0	0	2	3	0	5	1	7	1	2	0	0	2	1	1	3	2	4

Continued

Supplementary Table 1. Classification by the Leprosy Elimination Monitoring Tool of the leprosy elimination phase in Alagoas. 2001-2022

Leprosy Elimination Monitoring Tool

- **Phase 1 – up to transmission interruption**
- **Phase 2 – up to disease elimination**
- **Phase 3 – post-elimination surveillance**
- **Phase 4 – non-endemic status**
- **Sporadic cases in adults**
- **Sporadic cases in children**
- **Average of three cases over three consecutive years**

Municipality	Cases	2001	2002	2003	2004	2005	2006	2007	2008	2009	2010	2011	2012	2013	2014	2015	2016	2017	2018	2019	2020	2021	2022
Jacaré dos Homens	<15 years	0	0	0	0	0	0	0	0	0	0	0	0	0	0	0	0	0	0	0	0	0	0
	Adult	1	1	0	0	0	0	0	0	1	0	0	4	0	1	0	0	0	0	1	0	1	0
	Total	1	1	0	0	0	0	0	0	1	0	0	4	0	1	0	0	0	0	1	0	1	0
Jacuípe	<15 years	0	0	0	0	0	0	0	0	0	0	0	0	0	0	0	0	1	0	0	0	0	0
	Adult	0	0	0	0	1	0	0	0	0	0	0	0	1	0	0	0	0	0	0	0	1	3
	Total	0	0	0	0	1	0	0	0	0	0	0	0	1	0	0	0	1	0	0	0	1	3
Japaratinga	<15 years	0	0	0	0	0	0	0	0	0	0	0	0	0	0	0	0	0	0	0	0	0	0
	Adult	0	0	0	0	1	0	0	1	0	0	0	0	0	1	2	0	0	1	0	1	1	1
	Total	0	0	0	0	1	0	0	1	0	0	0	0	0	1	2	0	0	1	0	1	1	1
Jaramataia	<15 years	0	0	0	0	0	0	0	0	0	0	0	0	0	0	0	0	0	0	0	0	0	0
	Adult	0	2	1	3	3	2	0	3	0	4	4	2	0	3	0	3	0	0	0	1	1	1
	Total	0	2	1	3	3	2	0	3	0	4	4	2	0	3	0	3	0	0	0	1	1	1
Jequiá da Praia	<15 years	0	0	0	0	0	0	0	0	0	0	0	1	0	0	0	2	0	0	0	0	0	0
	Adult	0	0	4	1	0	1	1	1	2	1	0	1	2	1	0	0	0	1	1	1	0	0
	Total	0	0	4	1	0	1	1	1	2	1	0	2	2	1	0	2	0	1	1	1	0	0
Joaquim Gomes	<15 years	0	0	0	0	0	0	0	0	0	0	1	0	0	0	0	0	0	0	0	0	0	0
	Adult	1	1	0	0	3	1	0	0	0	0	0	3	0	1	1	0	0	0	0	1	1	0
	Total	1	1	0	0	3	1	0	0	0	0	1	3	0	1	1	0	0	0	0	1	1	0

Continued

Supplementary Table 1. Classification by the Leprosy Elimination Monitoring Tool of the leprosy elimination phase in Alagoas. 2001-2022

Leprosy Elimination Monitoring Tool	
	Phase 1 – up to transmission interruption
	Phase 2 – up to disease elimination
	Phase 3 – post-elimination surveillance
	Phase 4 – non-endemic status
	Sporadic cases in adults
	Sporadic cases in children
	Average of three cases over three consecutive years

Municipality	Cases	2001	2002	2003	2004	2005	2006	2007	2008	2009	2010	2011	2012	2013	2014	2015	2016	2017	2018	2019	2020	2021	2022
Jundiá	<15 years	0	0	0	0	0	1	0	0	0	0	0	0	0	0	0	0	0	0	0	0	0	0
	Adult	1	1	0	0	1	2	0	0	0	0	0	0	0	0	1	0	0	0	1	0	0	0
	Total	1	1	0	0	1	3	0	0	0	0	0	0	0	0	1	0	0	0	1	0	0	0
Junqueiro	<15 years	0	0	0	0	0	1	0	0	0	0	0	0	0	0	0	0	0	0	0	0	0	0
	Adult	1	4	0	1	4	0	1	2	1	5	3	2	1	0	0	1	1	0	0	0	1	2
	Total	1	4	0	1	4	1	1	2	1	5	3	2	1	0	0	1	1	0	0	0	1	2
Lagoa da Canoa	<15 years	0	0	0	0	0	0	0	0	0	1	0	0	0	0	0	0	0	0	0	0	0	0
	Adult	1	1	1	0	4	2	0	2	3	1	1	3	2	2	1	1	0	1	1	1	1	0
	Total	1	1	1	0	4	2	0	2	3	2	1	3	2	2	1	1	0	1	1	1	1	0
Limoeiro de Anadia	<15 years	0	0	0	0	0	0	0	0	0	0	0	0	1	0	0	0	1	0	0	0	0	0
	Adult	0	0	1	0	0	0	1	0	0	2	0	0	0	0	0	0	0	0	0	2	0	0
	Total	0	0	1	0	0	0	1	0	0	2	0	0	1	0	0	0	1	0	0	2	0	0
Maceió	<15 years	8	11	16	12	8	9	4	5	6	9	3	11	10	9	6	5	4	5	2	5	1	1
	Adult	139	166	218	150	141	153	135	142	132	110	106	120	110	90	108	85	85	103	78	56	63	74
	Total	147	177	234	162	149	162	139	147	138	119	109	131	120	99	114	90	89	108	80	61	64	75
Major Isidoro	<15 years	0	0	0	0	1	1	1	0	0	0	2	1	0	0	0	0	0	0	0	0	0	0
	Adult	2	1	1	5	5	4	4	3	4	2	9	4	1	2	0	1	4	0	2	2	0	4
	Total	2	1	1	5	6	5	5	3	4	2	11	5	1	2	0	1	4	0	2	2	0	4

Continued

Supplementary Table 1. Classification by the Leprosy Elimination Monitoring Tool of the leprosy elimination phase in Alagoas. 2001-2022

Leprosy Elimination Monitoring Tool

- **Phase 1 – up to transmission interruption**
- **Phase 2 – up to disease elimination**
- **Phase 3 – post-elimination surveillance**
- **Phase 4 – non-endemic status**
- **Sporadic cases in adults**
- **Sporadic cases in children**
- **Average of three cases over three consecutive years**

Municipality	Cases	2001	2002	2003	2004	2005	2006	2007	2008	2009	2010	2011	2012	2013	2014	2015	2016	2017	2018	2019	2020	2021	2022
Mar Vermelho	<15 years	0	0	0	0	0	0	0	0	0	0	0	0	0	0	0	0	0	0	0	0	0	0
	Adult	0	0	0	0	0	0	0	0	1	0	0	0	0	0	0	0	0	0	0	0	0	0
	Total	0	0	0	0	0	0	0	0	1	0	0	0	0	0	0	0	0	0	0	0	0	0
Maragogi	<15 years	0	0	0	2	0	0	0	0	1	1	3	0	0	0	0	0	0	1	0	0	0	0
	Adult	1	1	2	0	2	2	0	2	3	17	6	3	3	3	4	0	4	6	4	5	2	2
	Total	1	1	2	2	2	2	0	2	4	18	9	3	3	3	4	0	4	7	4	5	2	2
Maravilha	<15 years	0	0	0	0	0	0	0	0	0	0	0	0	0	0	0	0	0	0	0	0	0	0
	Adult	0	0	1	0	0	0	0	1	0	0	0	0	0	0	0	0	0	0	0	0	1	1
	Total	0	0	1	0	0	0	0	1	0	0	0	0	0	0	0	0	0	0	0	0	1	1
Marechal Deodoro	<15 years	0	1	0	0	0	1	0	0	0	1	0	0	1	1	1	2	0	0	0	0	0	0
	Adult	2	2	3	8	3	2	1	2	3	5	5	3	3	3	6	6	2	0	4	1	0	7
	Total	2	3	3	8	3	3	1	2	3	6	5	3	4	4	7	8	2	0	4	1	0	7
Maribondo	<15 years	0	0	0	0	0	0	0	0	0	0	0	0	0	0	0	0	0	0	0	0	0	0
	Adult	3	1	3	4	0	2	2	1	2	2	0	2	0	1	2	0	3	0	1	1	0	4
	Total	3	1	3	4	0	2	2	1	2	2	0	2	0	1	2	0	3	0	1	1	0	4
Mata Grande	<15 years	0	0	0	0	0	0	0	0	0	0	0	1	3	4	0	0	0	0	0	0	0	0
	Adult	0	0	1	1	1	2	1	4	2	4	3	5	5	1	2	1	0	1	0	0	1	1
	Total	0	0	1	1	1	2	1	4	2	4	3	6	8	5	2	1	0	1	0	0	1	1

Continued

Supplementary Table 1. Classification by the Leprosy Elimination Monitoring Tool of the leprosy elimination phase in Alagoas. 2001-2022

Leprosy Elimination Monitoring Tool	
	**Phase 1 – up to transmission interruption**
	**Phase 2 – up to disease elimination**
	**Phase 3 – post-elimination surveillance**
	**Phase 4 – non-endemic status**
	**Sporadic cases in adults**
	**Sporadic cases in children**
	**Average of three cases over three consecutive years**

Municipality	Cases	2001	2002	2003	2004	2005	2006	2007	2008	2009	2010	2011	2012	2013	2014	2015	2016	2017	2018	2019	2020	2021	2022
Matriz de Camaragibe	<15 years	0	0	0	0	0	0	0	0	0	0	0	0	0	0	0	0	0	0	0	0	0	0
	Adult	1	2	5	2	0	1	1	1	2	1	0	1	1	0	3	0	2	0	0	1	1	2
	Total	1	2	5	2	0	1	1	1	2	1	0	1	1	0	3	0	2	0	0	1	1	2
Messias	<15 years	0	0	0	0	0	0	0	0	0	0	0	1	0	0	0	0	0	0	0	0	0	0
	Adult	1	0	2	1	1	1	0	1	1	2	1	1	1	0	1	0	0	0	0	0	0	0
	Total	1	0	2	1	1	1	0	1	1	2	1	2	1	0	1	0	0	0	0	0	0	0
Minador do Negrão	<15 years	0	0	0	0	0	0	0	0	0	0	0	0	0	0	0	0	0	0	0	0	0	0
	Adult	0	1	0	0	1	0	0	0	0	0	0	0	0	0	0	0	0	0	0	0	0	1
	Total	0	1	0	0	1	0	0	0	0	0	0	0	0	0	0	0	0	0	0	0	0	1
Monteirópolis	<15 years	0	0	0	0	0	0	0	0	0	0	0	0	0	0	0	0	0	0	0	0	0	0
	Adult	0	0	0	0	0	0	0	0	2	0	0	0	0	0	0	0	1	0	1	1	0	2
	Total	0	0	0	0	0	0	0	0	2	0	0	0	0	0	0	0	1	0	1	1	0	2
Murici	<15 years	0	0	0	0	0	0	0	0	0	0	0	0	0	0	0	0	0	0	0	0	0	0
	Adult	1	3	1	0	1	1	1	1	0	1	3	1	4	3	3	1	4	5	5	0	2	1
	Total	1	3	1	0	1	1	1	1	0	1	3	1	4	3	3	1	4	5	5	0	2	1
Novo Lino	<15 years	0	0	0	0	0	0	0	0	0	0	0	0	0	0	0	0	0	0	0	0	0	0
	Adult	0	1	0	0	1	2	0	3	1	1	1	0	0	0	0	1	2	0	0	0	0	0
	Total	0	1	0	0	1	2	0	3	1	1	1	0	0	0	0	1	2	0	0	0	0	0

Continued

Supplementary Table 1. Classification by the Leprosy Elimination Monitoring Tool of the leprosy elimination phase in Alagoas. 2001-2022

Leprosy Elimination Monitoring Tool

- **Phase 1 – up to transmission interruption**
- **Phase 2 – up to disease elimination**
- **Phase 3 – post-elimination surveillance**
- **Phase 4 – non-endemic status**
- **Sporadic cases in adults**
- **Sporadic cases in children**
- **Average of three cases over three consecutive years**

Municipality	Cases	2001	2002	2003	2004	2005	2006	2007	2008	2009	2010	2011	2012	2013	2014	2015	2016	2017	2018	2019	2020	2021	2022
Olho d'Água das Flores	<15 years	1	0	0	0	0	0	0	0	0	0	0	0	2	1	0	0	2	0	0	0	0	0
	Adult	0	1	4	6	3	4	4	5	3	5	4	3	3	4	3	1	1	7	3	1	2	5
	Total	1	1	4	6	3	4	4	5	3	5	4	3	5	5	3	1	3	7	3	1	2	5
Olho d'Água do Casado	<15 years	0	0	0	0	1	0	0	0	0	0	0	0	0	0	0	0	0	0	0	0	0	0
	Adult	0	0	0	1	2	3	0	0	1	0	3	0	0	0	0	0	0	2	0	0	1	0
	Total	0	0	0	1	3	3	0	0	1	0	3	0	0	0	0	0	0	2	0	0	1	0
Olho d'Água Grande	<15 years	0	0	0	0	0	0	0	0	0	0	0	0	0	0	0	0	0	0	0	0	0	0
	Adult	0	0	0	0	0	0	1	0	0	0	0	0	0	0	0	0	0	0	0	0	0	0
	Total	0	0	0	0	0	0	1	0	0	0	0	0	0	0	0	0	0	0	0	0	0	0
Olivença	<15 years	0	0	0	0	0	0	0	0	0	0	0	0	0	0	0	0	0	0	0	0	0	0
	Adult	0	1	0	2	1	0	0	1	2	0	0	2	1	0	0	0	0	2	0	1	0	2
	Total	0	1	0	2	1	0	0	1	2	0	0	2	1	0	0	0	0	2	0	1	0	2
Ouro Branco	<15 years	0	0	0	0	0	0	0	0	0	0	0	1	0	0	0	0	0	0	0	0	0	0
	Adult	0	0	1	1	0	0	1	1	0	0	0	4	0	0	0	0	6	0	0	0	1	0
	Total	0	0	1	1	0	0	1	1	0	0	0	5	0	0	0	0	6	0	0	0	1	0
Palestina	<15 years	0	0	0	0	0	0	0	0	0	0	0	0	0	1	0	0	0	2	0	0	0	0
	Adult	0	0	0	0	0	0	0	0	0	0	0	1	1	4	0	1	2	3	2	2	2	10
	Total	0	0	0	0	0	0	0	0	0	0	0	1	1	5	0	1	2	5	2	2	2	10

Continued

Supplementary Table 1. Classification by the Leprosy Elimination Monitoring Tool of the leprosy elimination phase in Alagoas. 2001-2022

Leprosy Elimination Monitoring Tool

- **Phase 1 – up to transmission interruption**
- **Phase 2 – up to disease elimination**
- **Phase 3 – post-elimination surveillance**
- **Phase 4 – non-endemic status**
- **Sporadic cases in adults**
- **Sporadic cases in children**
- **Average of three cases over three consecutive years**

Municipality	Cases	2001	2002	2003	2004	2005	2006	2007	2008	2009	2010	2011	2012	2013	2014	2015	2016	2017	2018	2019	2020	2021	2022
Palmeira dos Índios	<15 years	0	0	0	0	0	1	1	0	0	1	1	0	0	0	0	0	0	0	1	0	0	0
	Adult	5	4	4	6	6	8	2	6	8	5	6	7	7	4	7	9	9	4	5	3	9	3
	Total	5	4	4	6	6	9	3	6	8	6	7	7	7	4	7	9	9	4	6	3	9	3
Pão de Açúcar	<15 years	0	0	1	0	0	0	0	0	0	0	0	0	0	1	1	0	0	0	3	2	0	2
	Adult	1	3	1	1	3	3	7	3	0	1	1	2	6	8	11	4	2	12	15	3	6	2
	Total	1	3	2	1	3	3	7	3	0	1	1	2	6	9	12	4	2	12	18	5	6	4
Pariconha	<15 years	0	0	0	0	0	0	0	1	1	0	0	0	0	0	0	0	1	0	0	0	0	0
	Adult	0	0	0	1	3	0	3	6	6	1	5	0	1	2	4	0	0	1	0	0	1	1
	Total	0	0	0	1	3	0	3	7	7	1	5	0	1	2	4	0	1	1	0	0	1	1
Paripueira	<15 years	0	0	0	0	0	0	0	0	0	0	0	0	0	0	0	0	0	0	0	0	0	0
	Adult	0	0	0	1	0	0	0	1	0	1	1	0	0	2	3	0	1	1	0	2	2	1
	Total	0	0	0	1	0	0	0	1	0	1	1	0	0	2	3	0	1	1	0	2	2	1
Passo de Camaragibe	<15 years	1	0	1	0	0	0	0	0	0	0	0	0	0	0	0	0	0	0	0	0	0	0
	Adult	0	1	0	3	3	0	0	0	1	1	0	0	0	1	1	0	1	0	0	0	0	0
	Total	1	1	1	3	3	0	0	0	1	1	0	0	0	1	1	0	1	0	0	0	0	0
Paulo Jacinto	<15 years	0	0	0	0	0	0	0	0	0	0	0	0	0	0	0	0	0	0	0	0	0	0
	Adult	0	0	0	0	2	0	0	0	2	0	1	0	0	1	1	0	0	0	0	0	1	0
	Total	0	0	0	0	2	0	0	0	2	0	1	0	0	1	1	0	0	0	0	0	1	0

Continued

Supplementary Table 1. Classification by the Leprosy Elimination Monitoring Tool of the leprosy elimination phase in Alagoas. 2001-2022

Leprosy Elimination Monitoring Tool	
	**Phase 1 – up to transmission interruption**
	**Phase 2 – up to disease elimination**
	**Phase 3 – post-elimination surveillance**
	**Phase 4 – non-endemic status**
	**Sporadic cases in adults**
	**Sporadic cases in children**
	**Average of three cases over three consecutive years**

Municipality	Cases	2001	2002	2003	2004	2005	2006	2007	2008	2009	2010	2011	2012	2013	2014	2015	2016	2017	2018	2019	2020	2021	2022
Penedo	<15 years	3	0	2	2	4	1	1	0	0	1	2	1	1	0	1	3	1	1	1	1	0	0
	Adult	19	19	14	20	14	13	15	16	27	15	17	11	8	11	5	17	12	14	8	6	9	7
	Total	22	19	16	22	18	14	16	16	27	16	19	12	9	11	6	20	13	15	9	7	9	7
Piaçabuçu	<15 years	0	0	0	0	0	1	0	2	0	0	0	0	0	0	1	1	0	0	0	0	0	0
	Adult	5	2	2	4	0	1	3	5	1	3	1	0	1	1	4	1	2	1	1	0	1	0
	Total	5	2	2	4	0	2	3	7	1	3	1	0	1	1	5	2	2	1	1	0	1	0
Pilar	<15 years	0	1	0	2	0	2	0	0	2	0	0	0	0	1	0	0	2	0	0	1	0	1
	Adult	0	3	11	11	16	11	17	9	12	5	8	7	8	7	6	9	8	6	7	16	5	9
	Total	0	4	11	13	16	13	17	9	14	5	8	7	8	8	6	9	10	6	7	17	5	10
Pindoba	<15 years	0	0	1	0	0	0	0	0	0	0	0	0	0	0	0	0	0	0	0	0	0	0
	Adult	0	0	0	0	0	0	0	0	0	0	0	0	0	0	0	0	0	0	0	0	0	0
	Total	0	0	1	0	0	0	0	0	0	0	0	0	0	0	0	0	0	0	0	0	0	0
Piranhas	<15 years	0	0	1	1	0	0	0	0	0	0	0	1	0	0	0	0	0	0	0	0	0	0
	Adult	2	0	2	4	2	10	3	3	1	1	0	2	2	4	2	1	3	1	2	0	4	2
	Total	2	0	3	5	2	10	3	3	1	1	0	3	2	4	2	1	3	1	2	0	4	2
Poço das Trincheiras	<15 years	0	0	0	0	0	0	0	0	0	0	0	0	0	0	0	0	0	0	0	0	0	0
	Adult	0	0	1	0	1	1	0	1	1	0	2	4	1	0	1	0	1	0	0	0	0	0
	Total	0	0	1	0	1	1	0	1	1	0	2	4	1	0	1	0	1	0	0	0	0	0

Continued

Supplementary Table 1. Classification by the Leprosy Elimination Monitoring Tool of the leprosy elimination phase in Alagoas. 2001-2022

Leprosy Elimination Monitoring Tool

- **Phase 1 – up to transmission interruption**
- **Phase 2 – up to disease elimination**
- **Phase 3 – post-elimination surveillance**
- **Phase 4 – non-endemic status**
- **Sporadic cases in adults**
- **Sporadic cases in children**
- **Average of three cases over three consecutive years**

Municipality	Cases	2001	2002	2003	2004	2005	2006	2007	2008	2009	2010	2011	2012	2013	2014	2015	2016	2017	2018	2019	2020	2021	2022
Porto Calvo	<15 years	1	0	0	1	0	0	2	0	0	0	0	0	0	0	0	0	0	0	0	0	0	0
	Adult	4	4	2	2	0	4	7	0	2	0	1	2	3	2	0	2	1	1	2	1	1	0
	Total	5	4	2	3	0	4	9	0	2	0	1	2	3	2	0	2	1	1	2	1	1	0
Porto de Pedras	<15 years	0	0	0	0	0	0	0	0	0	1	0	0	0	0	0	0	0	0	0	1	0	0
	Adult	0	0	0	0	2	0	0	0	1	2	0	3	0	1	0	0	1	0	0	0	0	0
	Total	0	0	0	0	2	0	0	0	1	3	0	3	0	1	0	0	1	0	0	1	0	0
Porto Real do Colégio	<15 years	0	0	0	0	0	0	0	0	0	0	0	0	0	0	0	0	0	0	0	0	0	0
	Adult	2	1	0	0	0	2	3	1	0	0	0	3	2	1	0	0	0	1	0	1	0	2
	Total	2	1	0	0	0	2	3	1	0	0	0	3	2	1	0	0	0	1	0	1	0	2
Quebrangulo	<15 years	0	0	0	0	0	0	0	0	0	0	0	0	0	0	0	0	0	0	0	0	0	0
	Adult	0	0	0	0	0	0	0	2	0	0	0	1	0	2	1	0	0	2	1	0	0	0
	Total	0	0	0	0	0	0	0	2	0	0	0	1	0	2	1	0	0	2	1	0	0	0
Rio Largo	<15 years	1	0	2	2	0	0	2	1	0	0	0	0	0	0	2	0	0	0	0	0	0	0
	Adult	7	7	7	7	9	6	13	9	5	13	6	11	11	7	12	11	13	5	3	7	9	6
	Total	8	7	9	9	9	6	15	10	5	13	6	11	11	7	14	11	13	5	3	7	9	6
Roteiro	<15 years	0	0	0	0	0	0	0	0	0	0	0	0	0	0	0	0	0	0	0	0	0	0
	Adult	0	0	0	0	0	0	0	0	0	0	1	0	0	0	0	0	0	0	1	0	1	0
	Total	0	0	0	0	0	0	0	0	0	0	1	0	0	0	0	0	0	0	1	0	1	0

Continued

Supplementary Table 1. Classification by the Leprosy Elimination Monitoring Tool of the leprosy elimination phase in Alagoas. 2001-2022

Leprosy Elimination Monitoring Tool	
	Phase 1 – up to transmission interruption
	Phase 2 – up to disease elimination
	Phase 3 – post-elimination surveillance
	Phase 4 – non-endemic status
	Sporadic cases in adults
	Sporadic cases in children
	Average of three cases over three consecutive years

Municipality	Cases	2001	2002	2003	2004	2005	2006	2007	2008	2009	2010	2011	2012	2013	2014	2015	2016	2017	2018	2019	2020	2021	2022
Santa Luzia do Norte	<15 years	0	0	0	0	0	1	0	0	0	0	0	0	0	1	0	0	1	0	0	0	0	0
	Adult	0	0	0	0	0	0	2	1	0	1	0	2	0	1	0	1	0	0	0	0	0	0
	Total	0	0	0	0	0	1	2	1	0	1	0	2	0	2	0	1	1	0	0	0	0	0
Santana do Ipanema	<15 years	0	2	2	1	0	2	0	1	2	0	2	3	2	1	5	0	9	5	1	0	0	2
	Adult	6	18	16	9	5	6	18	21	34	22	38	27	23	29	21	6	14	19	11	6	13	12
	Total	6	20	18	10	5	8	18	22	36	22	40	30	25	30	26	6	23	24	12	6	13	14
Santana do Mundaú	<15 years	0	0	0	0	0	0	0	0	0	0	0	0	0	0	0	0	0	0	0	0	0	0
	Adult	0	0	2	3	2	1	1	0	1	2	0	2	0	1	0	0	0	0	1	2	0	1
	Total	0	0	2	3	2	1	1	0	1	2	0	2	0	1	0	0	0	0	1	2	0	1
São Brás	<15 years	0	0	0	0	0	0	0	0	0	0	0	0	0	0	0	0	0	1	2	0	0	0
	Adult	2	0	0	1	0	3	3	1	0	0	0	0	2	1	0	0	1	0	1	2	1	1
	Total	2	0	0	1	0	3	3	1	0	0	0	0	2	1	0	0	1	1	3	2	1	1
São José da Laje	<15 years	0	0	0	0	0	0	0	0	0	0	0	0	0	0	1	0	1	0	0	0	0	0
	Adult	0	1	1	1	2	1	0	4	1	3	2	1	0	1	1	2	1	0	0	2	1	1
	Total	0	1	1	1	2	1	0	4	1	3	2	1	0	1	2	2	2	0	0	2	1	1
São José da Tapera	<15 years	0	0	1	0	0	0	0	0	0	0	0	0	0	2	0	0	0	2	2	0	1	1
	Adult	0	1	1	1	4	1	3	2	3	2	0	1	3	6	1	5	4	9	5	3	3	5
	Total	0	1	2	1	4	1	3	2	3	2	0	1	3	8	1	5	4	11	7	3	4	6

Continued

Supplementary Table 1. Classification by the Leprosy Elimination Monitoring Tool of the leprosy elimination phase in Alagoas. 2001-2022

Leprosy Elimination Monitoring Tool

- **Phase 1 – up to transmission interruption**
- **Phase 2 – up to disease elimination**
- **Phase 3 – post-elimination surveillance**
- **Phase 4 – non-endemic status**
- **Sporadic cases in adults**
- **Sporadic cases in children**
- **Average of three cases over three consecutive years**

Municipality	Cases	2001	2002	2003	2004	2005	2006	2007	2008	2009	2010	2011	2012	2013	2014	2015	2016	2017	2018	2019	2020	2021	2022
São Luís do Quitunde	<15 years	0	0	0	0	0	0	0	0	0	0	0	0	0	0	0	0	0	0	0	0	0	0
	Adult	0	1	1	1	0	2	0	1	1	0	0	0	0	1	0	0	1	0	0	1	0	1
	Total	0	1	1	1	0	2	0	1	1	0	0	0	0	1	0	0	1	0	0	1	0	1
São Miguel dos Campos	<15 years	0	1	1	1	3	1	0	0	0	0	0	0	0	0	0	0	0	1	0	0	0	0
	Adult	2	5	5	7	8	6	2	5	8	2	3	4	0	2	0	2	2	3	5	1	1	1
	Total	2	6	6	8	11	7	2	5	8	2	3	4	0	2	0	2	2	4	5	1	1	1
São Miguel dos Milagres	<15 years	2	2	0	0	0	0	0	0	0	0	0	0	0	0	0	0	0	0	0	0	0	0
	Adult	4	4	1	1	1	2	0	2	0	0	0	0	0	0	0	0	0	0	0	1	1	1
	Total	6	6	1	1	1	2	0	2	0	0	0	0	0	0	0	0	0	0	0	1	1	1
São Sebastião	<15 years	0	0	0	1	0	0	0	0	0	0	0	0	0	0	0	0	0	0	0	0	0	0
	Adult	1	4	2	2	2	2	4	0	0	0	1	2	0	2	2	1	0	1	1	1	0	3
	Total	1	4	2	3	2	2	4	0	0	0	1	2	0	2	2	1	0	1	1	1	0	3
Satuba	<15 years	0	0	0	0	0	0	0	0	0	1	0	0	0	0	0	0	0	0	0	0	1	0
	Adult	1	0	0	2	0	0	2	1	1	1	3	1	4	2	1	2	2	1	1	0	0	1
	Total	1	0	0	2	0	0	2	1	1	2	3	1	4	2	1	2	2	1	1	0	1	1
Senador Rui Palmeira	<15 years	0	0	0	0	0	0	0	0	0	0	0	0	0	0	0	0	0	0	0	0	0	0
	Adult	0	0	0	0	0	0	0	0	0	0	1	0	0	0	2	0	0	1	1	2	3	1
	Total	0	0	0	0	0	0	0	0	0	0	1	0	0	0	2	0	0	1	1	2	3	1

Continued

Supplementary Table 1. Classification by the Leprosy Elimination Monitoring Tool of the leprosy elimination phase in Alagoas. 2001-2022

Leprosy Elimination Monitoring Tool

- **Phase 1 – up to transmission interruption**
- **Phase 2 – up to disease elimination**
- **Phase 3 – post-elimination surveillance**
- **Phase 4 – non-endemic status**
- **Sporadic cases in adults**
- **Sporadic cases in children**
- **Average of three cases over three consecutive years**

Municipality	Cases	2001	2002	2003	2004	2005	2006	2007	2008	2009	2010	2011	2012	2013	2014	2015	2016	2017	2018	2019	2020	2021	2022
Tanque d'Arca	<15 years	0	0	0	0	0	0	0	0	0	0	0	0	0	0	0	0	0	0	0	0	0	0
	Adult	0	0	0	0	0	2	1	0	0	0	0	1	0	1	1	0	0	0	1	0	0	0
	Total	0	0	0	0	0	2	1	0	0	0	0	1	0	1	1	0	0	0	1	0	0	0
Taquarana	<15 years	0	0	0	0	0	0	0	0	0	0	0	0	0	0	1	0	0	0	0	0	1	0
	Adult	3	0	0	3	1	1	1	1	1	0	1	1	0	1	0	1	2	1	0	1	0	0
	Total	3	0	0	3	1	1	1	1	1	0	1	1	0	1	1	1	2	1	0	1	1	0
Teotônio Vilela	<15 years	0	1	1	0	0	1	0	0	1	0	1	0	0	1	0	0	0	0	0	0	1	0
	Adult	5	5	8	8	8	2	5	5	9	8	7	11	2	3	3	2	8	8	4	8	3	5
	Total	5	6	9	8	8	3	5	5	10	8	8	11	2	4	3	2	8	8	4	8	4	5
Traipu	<15 years	0	1	0	0	0	0	0	0	0	0	0	0	0	0	0	0	0	0	0	0	0	0
	Adult	2	4	1	1	0	1	0	1	1	0	0	0	1	1	0	0	1	0	0	0	2	0
	Total	2	5	1	1	0	1	0	1	1	0	0	0	1	1	0	0	1	0	0	0	2	0
União dos Palmares	<15 years	3	1	2	2	5	3	1	1	2	1	2	2	1	2	3	1	0	3	0	0	1	2
	Adult	21	26	32	30	26	27	22	11	14	12	21	19	20	10	13	4	14	11	11	7	15	9
	Total	24	27	34	32	31	30	23	12	16	13	23	21	21	12	16	5	14	14	11	7	16	11
Viçosa	<15 years	0	0	0	0	0	0	0	0	0	0	0	0	0	0	0	0	0	0	0	0	0	0
	Adult	0	2	0	0	2	1	1	1	1	2	2	1	1	1	1	2	0	4	2	0	1	1
	Total	0	2	0	0	2	1	1	1	1	2	2	1	1	1	1	2	0	4	2	0	1	1
