# Supplementary material for: Leprosy elimination phase in Alagoas, 2001-2022: an ecological study
Source: Epidemiol Serv Saude. 2025 Apr 11;34:e20240255. doi: 10.1590/S2237-96222024v34e20240255.en (PMC11998656; doi:10.1590/S2237-96222024v34e20240255.en)
Supplement: Translation

# Translation: Estágio de eliminação da hanseníase em Alagoas, 2001-2022: estudo ecológico

**Authors:** Lucas Vinícius de Lima, Gabriel Pavinati, Rayssa Gysele Teixeira da Silva, Itanielly Gomes Queiroz, Gustavo Laine Araújo de Oliveira, Sandra Maria Barbosa Durães, Miguel Angel Aragón López, Kleydson Bonfim Andrade Alves, Francisco Beraldi-Magalhães, Gabriela Tavares Magnabosco

**Affiliations:** 1 Universidade Estadual de Maringá, Programa de Pós-Graduação em Enfermagem, Maringá, PR, Brasil; 2 Secretaria de Estado da Saúde de Alagoas, Gerência de Vigilância e Controle de Doenças Transmissíveis, Maceió, AL, Brasil; 3 Ministério da Saúde, Coordenação-Geral de Vigilância das Doenças em Eliminação, Brasília, DF, Brasil; 4 Organização Pan-Americana da Saúde, Coordenação de Doenças Transmissíveis e Determinantes Ambientais da Saúde, Brasília, DF, Brasil; 5 Universidade do Estado do Amazonas, Fundação de Medicina Tropical Doutor Heitor Vieira Dourado, Manaus, AM, Brasil

**Keywords:** Hanseníase, Análise Espacial, Vigilância em Saúde Pública, Estudos Ecológicos, Estudos de Séries Temporais

## Abstract

**Objetivo:**

Analisar o cenário epidemiológico e o estágio de eliminação da hanseníase nos municípios de Alagoas, no período de 2001 a 2022.

**Métodos:**

Estudo ecológico com dados de vigilância provenientes do Sistema de Informação de Agravos de Notificação; utilizaram-se taxas de detecção e prevalência da doença, bem como frequências absolutas e relativas; realizaram-se análise de tendência por regressão joinpoint, aplicação dos índices de autocorrelação espacial de Moran e classificação dos municípios com a Leprosy Elimination Monitoring Tool (LEMT), proposta pela Organização Mundial da Saúde.

**Resultados:**

Entre 2010 e 2022, houve queda anual significativa de -2,89% (intervalo de confiança de 95% [IC95%] -5,65; -0,91) da taxa de prevalência, de -4,43% (IC95% -6,56; -2,20) da taxa de detecção na população geral e de -6,03% (IC95% -10,00; -1,46) da taxa de detecção na população menor de 15 anos; visualizaram-se clusters significativos (p-valor<0,050) do tipo alto-alto nos municípios de Jacaré dos Homens, Pão de Açúcar e Carneiros; evidenciou-se, pela LEMT, que, em 2022: 7 (6,8%) municípios estavam na fase 1 - até a interrupção da transmissão, 41 (40,2%) na fase 2 - até a eliminação da doença, 27 (26,5%) na fase 3 - vigilância pós-eliminação e 27 (26,5%) na fase 4 - estado não endêmico.

**Conclusão:**

Os dados apontaram para tendências de redução da carga de hanseníase no estado, embora alguns municípios persistam com taxas elevadas; o emprego da LEMT com dados secundários deve ser cauteloso, visto que a ausência de casos não implica na eliminação da doença em cenários de subnotificação e subdiagnóstico.

**Aspectos éticos**

A presente pesquisa respeitou os princípios éticos, obtendo os seguintes dados de aprovação:

A pesquisa atendeu aos princípios éticos preconizados pelo Conselho Nacional de Saúde, sendo dispensada de apreciação no Comitê de Ética em Pesquisa da Universidade Estadual de Maringá, por se basear em dados secundários, de domínio público e desidentificados, com base no art. 26, incisos III e V, da Resolução nº 674, de 6 de maio de 2022.

## Introdução

A hanseníase persiste como um desafio sobretudo para os países com menor nível socioeconômico e disponibilidade de recursos, a exemplo do Brasil ^1^ ^,^ ^2^. Embora tenham sido evidenciados progressos no processo de eliminação da hanseníase, especialmente pelas Estratégias Globais para Enfrentamento (2016-2020 e 2021-2030), propostas pela Organização Mundial da Saúde (OMS) ^2^, o Brasil ocupa a segunda posição no ranking mundial de novos casos da doença ^1^ ^,^ ^3^.

No Brasil, o Ministério da Saúde propôs as Estratégias Nacionais para Enfrentamento (2019-2022 e 2024-2030) ^1^ ^,^ ^4^. A proposta mais recente foi elaborada de forma participativa e visa, até 2030, à interrupção da transmissão em 99% dos municípios e à eliminação da doença em 75% deles ^4^. Para tanto, estabeleceram-se os pilares: aprimoramento da gestão dos programas de hanseníase, diagnóstico precoce por meio de busca ativa e assistência integral a pessoas acometidas pela doença ^4^.

Entre 2011 e 2021 foram registrados 309.638 novos casos de hanseníase no país, representando uma média anual de 28 mil casos ^5^. É válido salientar que a ocorrência da doença varia em função de fatores sociais, ambientais, econômicos e demográficos ^1^ ^,^ ^4^. No Brasil, em 2022, tal ocorrência foi particularmente mais concentrada nas regiões Norte, Nordeste e Centro-Oeste, com destaque para os estados do Mato Grosso e do Maranhão, que tiveram os maiores números de casos novos ^4^.

Estudo identificou clusters de altas taxas de detecção nos estados do Pará, Tocantins, Maranhão e Mato Grosso; por outro lado, os estados das regiões Sul e Sudeste concentraram o menor quantitativo de casos ^5^. Na região Nordeste, destaca-se o estado de Alagoas, com cerca de 250 novos casos ao ano ^6^. Embora não seja um estado com elevado número de casos, em comparação a outros da mesma região, ele apresenta o segundo menor índice de desenvolvimento humano dentre os estados brasileiros ^7^.

O estado alagoano visa à eliminação da hanseníase até 2030, em conformidade com as diretrizes nacionais e internacionais, contudo, persistem alguns obstáculos, como: fragilidade na notificação dos casos, baixa adesão das equipes de saúde da família para diagnóstico e tratamento, ausência de ações de educação em saúde, déficit de serviços e profissionais de saúde habilitados para diagnóstico, e persistência de ações discriminatórias e estigma para com as pessoas acometidas ^8^.

Em 2023, a OMS propôs definições, critérios e indicadores na Leprosy Elimination Monitoring Tool (LEMT), uma ferramenta para avaliar a interrupção da cadeia de transmissão e o estágio de eliminação da hanseníase ^9^. A LEMT promove uma forma padronizada de monitorizar detalhadamente o progresso em direção à interrupção da transmissão e à eliminação da doença, especialmente por seu caráter ascendente de coleta de evidências em relação aos marcos estabelecidos pela OMS ^9^.

No contexto internacional, a LEMT já foi empregada para a avaliação dos estágios de eliminação da hanseníase na Tailândia, conforme o exemplo apresentado pela OMS ^9^. Em busca realizada na PubMed Central, em 5 de outubro de 2024, com a estratégia Leprosy Elimination Monitoring Tool, não foram encontrados estudos sobre o uso da ferramenta. Nacionalmente, com a mesma estratégia na Biblioteca Virtual em Saúde, também não foram recuperados artigos científicos no escopo desta pesquisa.

Nesse sentido, considera-se que o primeiro passo para o alcance das metas de eliminação, propostas nacional e internacionalmente, até 2030 consiste na identificação de áreas prioritárias para avaliar e redirecionar intervenções, com foco na otimização de ações e repostas em saúde ^9^. Justifica-se a realização do presente estudo, cujo objetivo foi analisar o cenário epidemiológico e o estágio de eliminação da hanseníase nos municípios de Alagoas, Brasil, no período de 2001 a 2022.

## Métodos

### Delineamento e contexto 

Trata-se de estudo ecológico, com análise temporal e espacial com dados dos 102 municípios e do estado de Alagoas, localizado no Nordeste do Brasil. Alagoas possui área de 27.830,661 km2 e uma população de 3.127.683 pessoas; estima-se que o índice de desenvolvimento humano seja de 0,684 ^7^ ^,^ ^10^. Seus 102 municípios estão dispostos em dez regiões sanitárias, possibilitando a organização das atividades de diagnóstico e acompanhamento da hanseníase na atenção primária à saúde.

### População e variáveis

Na primeira etapa do estudo, a partir do painel, considerou-se o recorte temporal de 2010 a 2022 pela disponibilidade de dados na data de acesso, em 17 de novembro de 2023. Foram extraídos três indicadores calculados pelo Ministério da Saúde, sendo que, no denominador, foram utilizados os dados sobre os habitantes, de cada município, oriundos do Instituto Brasileiro de Geografia e Estatística. Os seguintes indicadores foram consultados, ano a ano, para estado e municípios ^3^:

taxa de prevalência dos casos em tratamento (por 10 mil habitantes): número de casos em tratamento em determinado local em 31 de dezembro do ano da avaliação, dividido pela população total residente no mesmo local e período, e o resultado multiplicado por 10 mil;

taxa de detecção de casos novos na população geral (por 100 mil habitantes): número de casos novos em determinado local e diagnosticados no ano da avaliação, dividido pela população total residente no mesmo local e período, e o resultado multiplicado por 100 mil; e

taxa de detecção de casos novos na população menor de 15 anos (por 100 mil habitantes): número de casos novos em menores de 15 anos em determinado local e diagnosticados no ano da avaliação, dividido pela população menor de 15 anos residente no mesmo local e período, e o resultado multiplicado por 100 mil.

Para a segunda etapa do estudo, consideraram-se os casos de hanseníase notificados como “caso novo”, com diagnóstico de 2001 a 2022 e que residiam nos municípios alagoanos. Foram excluídos os registros com “erro de diagnóstico”. Essa organização do banco permitiu a construção de duas variáveis, por município e ano: (i) número de casos novos de hanseníase na população com idade igual ou superior a 15 anos e (ii) número de casos novos de hanseníase na população menor de 15 anos.

### Fonte de dados e mensuração

Foram utilizados dados do Painel de Indicadores e Dados Básicos de Hanseníase nos Municípios Brasileiros, compilados e disponibilizados publicamente pelo Ministério da Saúde do Brasil e do Sistema Nacional de Informação de Agravos de Notificação (Sinan) (6), fornecidos pela Secretaria de Estado de Saúde de Alagoas. O painel foi utilizado para a primeira etapa do estudo (análise temporal e espacial). Nessa etapa, foram consultados os seguintes indicadores epidemiológicos da doença:

taxa de prevalência dos casos em tratamento: baixo (<1,00/10 mil habitantes), médio (1,00 a 4,99/10 mil habitantes), alto (5,00 a 9,99/10 mil habitantes), muito alto (10,00 a 19,99/10 mil habitantes) e hiperendêmico (≥20,00/10 mil habitantes);

taxa de detecção de casos novos na população geral: baixo (<2,00/100 mil habitantes), médio (2,00 a 9,99/100 mil habitantes), alto (10,00 a 19,99/100 mil habitantes), muito alto (20,00 a 39,99/100 mil habitantes) e hiperendêmico (≥40,00/100 mil habitantes); e

taxa de detecção de casos novos na população menor de 15 anos: baixo (<0,50/100 mil habitantes), médio (0,50 a 2,49/100 mil habitantes), alto (2,50 a 4,99/100 mil habitantes), muito alto (5,00 a 9,99/100 mil habitantes) e hiperendêmico (≥10,00/100 mil habitantes).

A base do Sinan foi empregada para a segunda etapa (análise do estágio de eliminação). Cabe pontuar que os dados provenientes do Sinan são utilizados para a alimentação do painel de indicadores, em consonância com o processo ascendente de envio de dados. No entanto, no painel constam indicadores já calculados pelo Ministério da Saúde de interesse ao estudo, em sua primeira etapa. Já no segundo momento do estudo, o período disponível no painel não era compatível com a aplicação da LEMT.

A LEMT é uma ferramenta proposta pela OMS para o monitoramento padronizado do estágio de eliminação da hanseníase, organizado em quatro fases sequenciais. A implementação da ferramenta a nível subnacional estimula um processo ascendente no qual cada uma das áreas administrativas busca individualmente o alcance dos marcos estabelecidos. Para fins de operacionalização da LEMT, foram consideradas as seguintes etapas de avaliação dos municípios ^9^:

fase 1 - até a interrupção da transmissão: o município permanece até não haver casos novos autóctones na população menor de 15 anos por cinco anos consecutivos;

fase 2 - até a eliminação da doença: o município permanece até não haver casos novos autóctones na população com idade igual ou superior a 15 anos por três anos consecutivos;

fase 3 - vigilância pós-eliminação: o município permanece até não haver casos novos autóctones por, no mínimo, dez anos consecutivos; e

fase 4 - estado não endêmico: o município permanece após sustentar a ausência de casos novos autóctones preconizada na fase anterior.

Ao considerar o conceito epidemiológico de casos autóctones, entende-se que são aqueles em que é possível estabelecer o vínculo de origem da transmissão no mesmo local em que aconteceram, ou seja, referem-se a pessoas que adquiriram a doença em seu território de residência. Devido à impossibilidade de se obter essa informação de forma precisa com dados secundários do Sinan, neste estudo, foram considerados autóctones os casos registrados na população residente no município.

Os casos esporádicos nas fases 2 a 4 precisariam ser individualmente investigados quanto à transmissão, dada a possibilidade de se estabelecer vínculo no local, ou seja, de ser autóctone; diante disso, o município deveria retornar ao estágio anterior. Ainda, a partir da fase 2, um município poderia regredir na ocorrência de três ou mais casos novos, em média, por três anos consecutivos ^9^. Neste estudo, somente o segundo critério foi considerado para reverter o estágio devido à natureza dos dados secundários.

### Métodos estatísticos

Inicialmente, realizou-se a análise de tendência das séries temporais, de 2010 a 2022, das taxas de detecção e de prevalência da hanseníase no estado. Empregou-se o modelo de regressão por pontos de inflexão no Joinpoint Regression Program, versão 5.0.2, tendo-se a transformação logarítmica das taxas como a variável dependente e os anos como a variável independente. Fez-se o ajuste de autocorrelação de primeira ordem e, devido à logaritmização, considerou-se o pressuposto da homoscedasticidade ^11^.

Os modelos finais foram escolhidos pelo menor valor do critério de informação bayesiano ponderado. Pelo método quantil empírico, calcularam-se as variações percentuais anuais (VPA) - mudança das taxas nos joinpoints; as variações percentuais anuais médias (VPAM) - médias geométricas das VPA; e os intervalos de confiança de 95% (IC95%). As VPA/VPAM positivas e negativas, cujo IC95% não incluísse o valor nulo, indicaram tendência de incremento e declínio, respectivamente ^11^.

Ainda na primeira etapa, as taxas anuais de prevalência e de detecção, entre 2010 e 2022, para cada município alagoano foram agrupadas por média aritmética para minimizar as flutuações aleatórias. O shapefile com os limites municipais e regionais, obtido publicamente, serviu à análise no software QGIS, versão 3.34.0. A partir disso, foram construídos mapas coropléticos para a distribuição espacial, segundo as respectivas classificações em parâmetros de endemicidade ^3^.

Aplicou-se o índice de Moran global (I), determinando a relação (inversa ou direta) da taxa média com ela mesma nas regiões vizinhas em um parâmetro: quanto mais perto de 1,00, mais forte a autocorrelação. Empregou-se a vizinhança do tipo rainha de primeira ordem e avaliou-se a significância pelo teste com 999 permutações (p-valor<0,050) no GeoDa, versão 1.22. Em seguida, calculou-se o índice de Moran local (Ii) para identificar os valores municipais, apresentando-os em agrupamentos ^12^:

alto-alto: município com alta taxa, cercado por municípios com altas taxas;

baixo-baixo: município com baixa taxa, cercado por municípios com baixas taxas;

alto-baixo: município com alta taxa, cercado por municípios com baixas taxas; e

baixo-alto: município com baixa taxa, cercado por municípios com altas taxas.

Na segunda etapa, utilizando-se os dados de 2001 a 2022, realizou-se a categorização dos municípios pela LEMT ^9^. Os resultados foram apresentados em quatro mapas temáticos, considerando-se intervalos temporais intencionalmente similares, sendo eles: 2006, 2011, 2016 e 2022. Como o primeiro marco (fase 1) requer, no mínimo, cinco anos, o período inicial de apresentação dos mapas coropléticos foi 2006. A partir disso, foram definidos, pelos autores, os demais recortes para apresentação.

## Resultados

De 2010 a 2022, registrou-se tendência decrescente de -2,89% (IC95% -5,65; -0,91) ao ano na prevalência da hanseníase em Alagoas, com queda mais acentuada entre 2010 e 2015 (VPA: -12,86; IC95% -25,33; -6,98). Notaram-se tendências decrescentes de -4,43% (IC95% -6,56; -2,20) ao ano na taxa de detecção de casos novos de hanseníase na população geral, bem como de -6,03% (IC95% -10,00; -1,46) ao ano na taxa de detecção na população menor de 15 anos (Tabela 1).

**Tabela 1:** Variações percentuais anuais (VPA), variações percentuais anuais médias (VPAM) e intervalos de confiança de 95% (IC95%) das taxas de prevalência na população geral, detecção na população geral e detecção na população menor de 15 anos em Alagoas. 2010-2022 (n=4.527)

Indicador	Período	VPA (IC95%)	Tendência	VPAM (IC95%)	Tendência
Prevalência	2010-2015	-12,86 (-25,33; -6,98)	Decrescente	-2,89 (-5,65; -0,91)	Decrescente
	2015-2018	14,90 (-3,65; 24,14)	Estacionária		
	2018-2022	-1,99 (-20,39; -5,85)	Decrescente		
Detecção	2010-2022	-4,43 (-6,56; -2,20)	Decrescente	-4,43 (-6,56; -2,20)	Decrescente
Detecção (<15 anos)	2010-2018	-1,36 (-5,68; 25,24)	Estacionária	-6,03 (-10,00; -1,46)	Decrescente
	2018-2022	-14,71 (-38,25; -5,23)	Decrescente		

Maiores taxas médias de prevalência para os municípios de Palestina (6,06/10 mil habitantes), Santana do Ipanema (2,95/10 mil habitantes) e Delmiro Gouveia (2,43/10 mil habitantes) (Figura 1A) foram observadas. As taxas de detecção de casos novos mais elevadas também foram visualizadas nos municípios de Palestina (49,98/100 mil habitantes e 16,59/100 mil habitantes) e Santana do Ipanema (42,50/100 mil habitantes e 14,98/100 mil habitantes) (Figuras 1B e 1C; Tabela 2).

**Figura 1:** Classificação endêmica das taxas médias de prevalência (A), detecção na população geral (B) e detecção na população menor de 15 anos (C) da hanseníase em Alagoas. 2010-2022 (n=4.527)

**Tabela 2:** Taxas municipais de prevalência na população geral (por 10 mil habitantes), detecção na população geral (por 100 mil habitantes) e detecção na população menor de 15 anos (por 100 mil habitantes) em Alagoas. 2010-2022 (n=4.527)

Município	Taxas médias		
	Prevalência	Detecção	Detecção (<15 anos)
Água Branca	0,46	4,56	2,30
Anadia	0,56	7,80	4,85
Arapiraca	1,18	13,21	1,89
Atalaia	0,50	7,33	2,11
Barra de Santo Antônio	0,84	7,45	3,45
Barra de São Miguel	2,17	31,27	6,67
Batalha	0,26	3,83	0,00
Belém	0,87	3,62	8,46
Belo Monte	0,00	0,00	0,00
Boca da Mata	0,24	3,43	0,00
Branquinha	1,01	13,08	2,19
Cacimbinhas	2,20	18,60	5,10
Cajueiro	0,47	14,79	7,73
Campestre	0,00	2,21	0,00
Campo Alegre	0,37	3,96	0,46
Campo Grande	0,26	1,75	0,00
Canapi	0,43	3,44	1,28
Capela	0,53	4,98	0,00
Carneiros	0,85	10,97	0,00
Chã Preta	0,42	4,15	0,00
Coité do Nóia	0,64	10,55	11,29
Colônia Leopoldina	0,29	5,21	1,05
Coqueiro Seco	1,20	16,07	4,68
Coruripe	1,16	15,09	3,35
Craíbas	0,87	9,40	0,99
Delmiro Gouveia	2,43	26,42	2,86
Dois Riachos	0,55	7,59	0,00
Estrela de Alagoas	0,81	8,97	1,53
Feira Grande	0,72	4,51	0,00
Feliz Deserto	0,67	5,07	0,00
Flexeiras	0,06	4,77	2,17
Girau do Ponciano	0,08	3,10	0,00
Ibateguara	0,41	2,52	0,00
Igaci	0,39	2,41	0,00
Igreja Nova	0,25	3,54	4,81
Inhapi	1,30	10,45	0,00
Jacaré dos Homens	1,02	10,10	0,00
Jacuípe	0,80	8,66	3,87
Japaratinga	1,18	6,35	0,00
Jaramataia	2,04	28,68	0,00
Jequiá da Praia	0,41	6,48	6,78
Joaquim Gomes	0,51	2,63	0,95
Jundiá	1,84	3,66	0,00
Junqueiro	0,22	3,79	0,00
Lagoa da Canoa	0,59	5,04	1,28
Limoeiro de Anadia	0,24	1,35	1,14
Maceió	0,78	9,73	2,10
Major Isidoro	1,50	13,61	2,71
Maragogi	1,11	15,11	5,46
Maravilha	0,58	2,46	0,00
Marechal Deodoro	0,74	8,13	3,31
Maribondo	0,97	9,06	0,00
Mar Vermelho	0,00	0,00	0,00
Mata Grande	0,89	7,08	6,29
Matriz de Camaragibe	0,22	3,75	0,00
Messias	0,24	3,68	2,96
Minador do Negrão	0,16	1,59	0,00
Monteirópolis	0,75	4,28	0,00
Murici	0,63	9,05	0,00
Novo Lino	0,61	3,06	0,00
Olho d'Água das Flores	1,80	16,96	6,29
Olho d'Água do Casado	0,60	6,83	0,00
Olho d'Água Grande	0,00	0,00	0,00
Olivença	0,54	6,13	0,00
Ouro Branco	0,68	7,48	2,21
Palestina	6,06	49,98	16,59
Palmeira dos Índios	0,67	7,79	1,25
Pão de Açúcar	1,75	25,94	9,57
Pariconha	0,29	12,43	2,62
Paripueira	0,89	7,21	0,00
Passo de Camaragibe	0,25	2,50	0,00
Paulo Jacinto	0,10	4,06	0,00
Penedo	1,22	17,86	5,12
Piaçabuçu	0,74	7,30	3,08
Pilar	1,83	22,45	3,39
Pindoba	0,00	0,00	0,00
Piranhas	0,66	7,16	0,00
Poço das Trincheiras	0,50	7,14	0,00
Porto Calvo	0,83	4,00	0,00
Porto de Pedras	0,80	6,40	2,67
Porto Real do Colégio	0,38	3,89	0,00
Quebrangulo	0,47	4,68	0,00
Rio Largo	1,22	12,17	1,11
Roteiro	0,92	3,47	0,00
Santa Luzia do Norte	0,53	8,54	7,88
Santana do Ipanema	2,95	42,50	14,98
Santana do Mundaú	0,49	5,59	0,00
São Brás	1,88	13,26	13,68
São José da Laje	0,89	4,59	2,25
São José da Tapera	0,72	13,45	6,27
São Luís do Quitunde	0,09	0,91	0,00
São Miguel dos Campos	0,27	3,27	0,49
São Miguel dos Milagres	0,48	3,80	0,00
São Sebastião	0,25	3,24	0,00
Satuba	0,96	11,17	4,33
Senador Rui Palmeira	0,97	7,92	0,00
Tanque d'Arca	0,25	3,71	0,00
Taquarana	0,47	4,26	3,03
Teotônio Vilela	0,97	13,43	1,71
Traipu	0,48	1,94	0,00
União dos Palmares	1,33	22,14	7,50
Viçosa	0,74	4,77	0,00

Pelo indicador de Moran global, visualizou-se ausência de autocorrelação para as taxas de prevalência (I: -0,01; p-valor 0,483) e de detecção na população geral (I: 0,01; p-valor 0,297) e menor de 15 anos (I: -0,07; p-valor 0,146). Ao aplicar o indicador local, foram visualizados aglomerados do tipo alto-alto para os municípios de Jacaré dos Homens e Pão de Açúcar para a taxa de prevalência (Figura 2A); e de Carneiros e Jacaré dos Homens para a taxa de detecção na população geral (Figura 2B).

**Figura 2:** Autocorrelação espacial das taxas médias de prevalência (A), detecção na população geral (B) e detecção na população menor de 15 anos (C) da hanseníase em Alagoas. 2010-2022 (n=4.527)

A categorização dos municípios pelos estágios de eliminação foi apresentada nos mapas referentes a 2006 (Figura 3A), 2011 (Figura 3B), 2016 (Figura 3C) e 2022 (Figura 3D). Identificou-se que, ao final do período analisado: 7 (6,8%) municípios estavam na fase 1; 41 (40,2%) estavam na fase 2; 27 (26,5%) estavam na fase 3; e 27 (26,5%) estavam na fase 4. Os dados de cada município podem ser visualizados individualmente na planilha da LEMT (Tabela Suplementar 1).

**Figura 3:** Classificação pela Leprosy Elimination Monitoring Tool do estágio de eliminação da hanseníase em Alagoas. 2006 (A), 2011 (B), 2016 (C) e 2022 (D)

## Discussão

O estado de Alagoas está empenhado no alcance das metas de eliminação da hanseníase como problema de saúde pública até 2030, apresentando tendência decrescente nas taxas de prevalência, de detecção na população geral e de detecção na população menor de 15 anos referentes ao período de 2010 a 2022. Dentre os municípios avaliados entre 2001 e 2022, destacaram-se 28 que se encontraram em estado de vigilância pós-eliminação da doença e 27 que foram classificados no estado não endêmico.

A redução da magnitude e da incidência da hanseníase pode indicar o fortalecimento de estratégias, tais como: aumento do diagnóstico precoce, com descentralização à atenção primária; promoção de ações educativas de prevenção junto à população; aumento da cobertura das ações em saúde da família, possibilitando a identificação precoce de pessoas em risco; e fortalecimento da busca ativa, principalmente entre contatos sociais e familiares de pessoas acometidas ^13^ ^-^ ^18^.

Embora a tendência de declínio dos indicadores da hanseníase esteja consoante a outros estudos nacionais, a doença apresenta altas taxas de detecção em regiões de pobreza e desigualdade social ^13^ ^-^ ^15^ ^,^ ^18^. Estudos ecológicos revelaram que as piores condições de vida da população, como baixa renda domiciliar per capita, baixo nível educacional e elevado número de moradores por domicílio, funcionaram como barreiras para acesso ao diagnóstico e aumentaram o risco de adoecimento pela hanseníase ^19^ ^-^ ^21^.

No estado alagoano, mesmo com a redução dos indicadores de prevalência e detecção, alguns municípios persistiram com características hiperendêmicas, como Palestina, Santana do Ipanema e Delmiro Gouveia. Esses municípios apresentaram, em 2010, os seguintes índices de desenvolvimento humano: 0,558, 0,591 e 0,612 ^7^. Além disso, o índice médio de Gini para esses municípios foi de 0,573, superando o valor para o estado, de 0,526, o que indica maior desigualdade na distribuição de renda ^7^.

Ademais, os municípios de Jacaré dos Homens, Pão de Açúcar e Carneiros concentraram bolsões de maior carga da hanseníase, conforme a avaliação de autocorrelação. Esses municípios estão localizados na nona região sanitária, o que sugere que tal área parece congregar a maior quantidade de municípios com altas taxas de detecção e prevalência. Isso sinaliza a necessidade de ações direcionadas para esses territórios, visando otimizar a resposta estadual de enfrentamento.

Em 2015, as projeções apontavam para o alcance das metas de eliminação até 2020 no Brasil, na Índia e na Indonésia. Todavia, a hanseníase continuaria como um problema nas regiões endêmicas desses países ^22^, principalmente pela dificuldade de priorização de recursos, deixando as populações mais vulneráveis em risco acrescido ^23^. No Brasil, em coorte com 23.899.942 pessoas, níveis reduzidos de renda e educação foram associados a um aumento de até duas vezes na incidência da doença ^24^.

Em contrapartida, locais com melhor acesso aos serviços de saúde podem apresentar maiores taxas de detecção dos casos de hanseníase ^25^. Dessa forma, entende-se que considerar as questões socioeconômicas e programáticas, especialmente por estudos que permitam identificar as particularidades locorregionais associadas ao contexto epidemiológico, pode auxiliar no reconhecimento de áreas prioritárias para a avaliação das estratégias/recomendações e para o direcionamento de novas intervenções ^26^ ^,^ ^27^.

Apesar de baixas taxas de prevalência e detecção de casos indicarem melhor progresso no controle da doença em algumas regiões, é premente destacar que a melhoria do acesso aos serviços de saúde constitui um importante fator associado ao aumento da taxa de detecção da hanseníase ^25^. Assim, a ausência de casos de hanseníase nos municípios em eliminação precisa ser cautelosamente compreendida, pois poderia resultar de um sistema de saúde frágil no que se refere à detecção/notificação da doença.

Embora a LEMT permita a ocorrência de casos novos esporádicos nas fases 3 e 4 ^9^, deve-se proceder à análise individual em cada situação, visto que aspectos como o grau de incapacidade física no diagnóstico e a avaliação de contatos para identificação de vínculo epidemiológico precisam ser considerados como potenciais fatores de confusão na conceituação de “esporádico”. Portanto, a articulação do nível estadual com os municípios é indispensável para que se consiga obter maior confiança no uso da LEMT.

Não obstante, ressalta-se que a avaliação dos estágios de eliminação da hanseníase deste estudo incluiu dois parâmetros diferentes ^9^. Isso sugere que os municípios em fases mais avançadas podem, de fato, apresentar um progresso efetivo no controle da doença. No entanto, é importante frisar a necessidade de fortalecimento dos sistemas de vigilância das doenças - em especial do Sinan -, que são cruciais para monitorar as tendências e os padrões dos casos de hanseníase por dados secundários ^28^.

Ainda em relação à eliminação da hanseníase, para além da redução do número de casos, preconizam-se o enfrentamento da discriminação e a prevenção das incapacidades ^29^. Os domínios físico, psicológico e social das pessoas acometidas são os mais comprometidos em sua qualidade de vida ^30^. Desse modo, não obstante às questões epidemiológicas destacadas neste estudo, é preciso avaliar o contexto de vida nos diferentes locais do país para monitorar a resposta de eliminação.

São limitações desta pesquisa: uso de dados secundários, que está sujeito à subnotificação dos casos; emprego de dados nacionais, visto que a base do painel de indicadores pode diferir das bases estaduais devido ao processo de qualificação; avaliação concomitante ao período da covid-19, que pode ter prejudicado a qualidade da detecção/notificação entre 2020 e 2022 ^5^; e impossibilidade de avaliação individual dos casos autóctones/esporádicos pelo uso exclusivo de dados secundários.

As descobertas indicaram uma redução significativa na prevalência e detecção de hanseníase em Alagoas, sugerindo o fortalecimento de estratégias de saúde, como diagnóstico precoce e busca ativa. No entanto, a persistência de áreas hiperendêmicas, especialmente em regiões de desigualdade social, destaca a necessidade de intervenções focadas. Essas ações devem priorizar o acesso aos serviços de saúde, a vigilância e o combate à discriminação, essenciais para alcançar a eliminação.

Ademais, o uso isolado da LEMT deve ser cauteloso, pois a ausência de casos não implica na eliminação nos territórios; nestas situações, deve-se considerar parâmetros de subdiagnóstico e subnotificação, como alto grau de incapacidade física no diagnóstico, não realização de busca ativa e debilidade dos serviços de testagem e vigilância. Além disso, os casos autóctones e esporádicos precisam ser analisados individualmente, verificando-se o vínculo epidemiológico para avaliar a transmissão comunitária.

Destaca-se que o processo de validação de métodos e resultados junto a profissionais de saúde e gestores federais e estaduais foi importante para o entendimento de determinadas dinâmicas municipais que fugiram do padrão esperado no processo de eliminação da doença. Nesse sentido, o uso da LEMT deve considerar o percurso do nível micro ao macro, pois essa articulação pode potencializar o emprego da ferramenta e qualificar a interpretação dos resultados de cada região.

## Data Availability

Os bancos de dados utilizados na pesquisa estão disponíveis em https://datasus.saude.gov.br/acesso-a-informacao/casos-de-hanseniase-desde-2001-sinan/ e http://indicadoreshanseniase.aids.gov.br/.
